# Human umbilical cord mesenchymal stem cells conditioned medium exerts anti-tumor effects on KGN cells in a cell density-dependent manner through activation of the Hippo pathway

**DOI:** 10.1186/s13287-023-03273-z

**Published:** 2023-03-20

**Authors:** Wenjing Wan, Yuyang Miao, Yuwei Niu, Kunyuan Zhu, Yingwan Ma, Menghao Pan, Baohua Ma, Qiang Wei

**Affiliations:** 1 Key Laboratory of Animal Biotechnology, Ministry of Agriculture, Northwest A and F University, Yangling, 712100 Shaanxi China; 2 College of Veterinary Medicine, Northwest A and F University, Yangling, 712100 Shaanxi China

**Keywords:** Human umbilical cord mesenchymal stem cells, Conditioned medium, Granulosa cell tumor, Cell density, Contact inhibition, Hippo pathway

## Abstract

**Background:**

The conditioned medium from human umbilical cord mesenchymal stem cells (UCMSCs-CM) provides a new cell-free therapy for tumors due to its unique secretome. However, there are many contradictory reports about the effect of UCMSCs-CM on tumor cells. The loss of contact inhibition is a common characteristic of tumor cells. A relationship between the effect of UCMSCs-CM on tumor cells and contact inhibition in tumor cells is rarely concerned. Whether the effect of UCMSCs-CM on tumor cells is affected by cell density? Here, we explored the effect of UCMSCs-CM on granulosa tumor cell line (KGN) cells at low or high density.

**Methods:**

Growth curve and CCK8 assay were used to assess cell proliferation and viability. Scratch wound and matrigel invasion assay were implicated to detect cell motility of KGN cells. UCMSCs-CM effects on cell cycle, apoptosis and pathway-related proteins were investigated by flow cytometry, TUNEL assay, western blot and immunofluorescence analysis respectively.

**Results:**

In growth curve analysis, before KGN cells proliferated into confluence, UCMSCs-CM had no effect on cell proliferation. However, once the cells proliferate to contact each other, UCMSCs-CM significantly inhibited proliferation. Meanwhile, when KGN cells were implanted at high density, UCMSCs-CM could induce cell cycle arrest at G1 phase, inhibit cell migration, invasion and promote apoptosis. While it had no similar effect on KGN cells implanted at low density. In mechanism, the UCMSCs-CM treatment activated the Hippo pathway when KGN cells were implanted at high density. Consistently, the MST1/2 inhibitor, XMU-MP-1, inhibited the activation of the Hippo pathway induced by UCMSCs-CM treatment and accordingly declined the anti-tumor effect of UCMSCs-CM on KGN cells.

**Conclusions:**

The effect of UCMSCs-CM on tumor cells is affected by cell density. UCMSCs-CM exerted anti-tumor effect on KGN cells by activating Hippo pathway to restore contact inhibition. Our results suggest that UCMSCs-CM is a promising therapeutic candidate for GCT treatment.

**Supplementary Information:**

The online version contains supplementary material available at 10.1186/s13287-023-03273-z.

## Introduction

Granulosa cell tumor (GCT) is a rare and severe sex cord stromal ovarian tumor, accounting for 5–8% of all ovarian malignancies [1]. GCTs are associated with significant risk of recurrence [2], which is often nonresponsive to conventional chemotherapies, and 80% of these recurrent cases succumb to their disease [1, 3, 4]. To date, there is no standard treatment for patients with recurrent GCT [5].

Umbilical cord mesenchymal stem cells (UCMSCs) are a promising tool in cell therapies due to their multi-potency, self-renewal and immunomodulatory properties [6]. These characteristics endow UCMSCs with potential for application in the novel tumor therapies [7]. In recent years, because of unique secretome and exosomes, the conditioned medium and cell lysate of UCMSCs were used of cell-free tumor therapy [8]. UCMSCs conditioned medium (CM) contains numbers of special exosomes, growth agents, and cytokines. However, the anti-cancer property of UCMSCs-CM remains largely elusive. The human UCMSCs extracts mediated the inhibition of the proliferation of ovarian cancer cells in vitro [9, 10], UCMSCs-CM inhibited the growth of mammary carcinoma, osteosarcoma, bladder cancer, and lymphoma cells in vitro and in vivo [10–12]. Moreover, UCMSCs-CM suppresses breast cancer cells growth and sensitizes cancer cells to radiotherapy through inhibition of the Stat3 signaling pathway [13]. On the other hand, it is reported that UCMSCs-CM promoted the proliferation and migration of glioblastoma cells, MDA-MB-231 cells, and some subtypes of lung cancer stem cells [14–16]. These contradictory results are usually attributed to the difference in sensibility of various tumor cell types.

The loss of contact inhibition or density-dependent inhibition is one of the key events in oncogenesis [17, 18]. Although there have been many reports about the effect of UCMSCs-CM on tumor cells, a correlation between these effects and contact inhibition on tumor cells is rarely concerned. The Hippo pathway is highly sensitive to cell density and mediate contact inhibition of growth [19, 20]. YAP1 (yes-associated protein 1), as a transcriptional coactivator of pro-proliferation and pro-tumor genes [21, 22], is a main downstream effector of the Hippo pathway [23]. It modulates many of the biological phenotypes of cancer cells, including cell proliferation, invasion and metastasis [24]. Higher YAP1 expression was significantly associated with poorer overall survival and disease-free survival in adrenocortical carcinoma (ACC), brain lower grade glioma (LGG), and pancreatic adenocarcinoma (PAAD) [25]. Notably, evidence also links the Hippo pathway to GCT with YAP being over-activated, which regulated of GCT cell proliferation and migration [26].

In the present study, we evaluated the effects of the UCMSCs-CM against the KGN cells at low and high density in vitro. Our study revealed a new mechanism that UCMSCs-CM inhibited the malignant phenotype of KGN cells related to cell density through activating Hippo pathway. The results obtained supported the utility of UCMSCs-CM as a candidate therapeutic agent for GCT.

## Materials and methods

### Isolation, culture and identification of hUCMSCs

Human umbilical cords were obtained from mothers who had given birth at Shaanxi maternal and Child Health Hospital and were used in accordance with the ethical guidelines and accepted human studies protocols at Northwest A&F University. Written informed consent forms were obtained from the healthy umbilical cord donors. HUCMSCs were prepared as previously described [27]. The surface markers including positive markers CD44, CD29, CD90, and CD105 and negative markers CD34, CD45 of hUCMSCs were analyzed by flow cytometry and CellQuest software (Becton Dickinson). The antibodies against the above surface markers were purchased from BD Biosciences, USA. The induced differentiation of hUCMSCs was conducted in osteogenic (Gibco, A1007201, USA), adipogenic (Gibco, A1007001, USA), or chondrogenic differentiation medium (Gibco, A1007101, USA) according to manufacturer's instructions. The microscopy images were acquired at 72 × 72 dpi resolution using an inverted microscope (Olympus, DP80, Japan). There was no any processing to enhance the resolution of the microscopy images.

### Cell culture

Human granulosa-like tumor cell line KGN was obtained from Fenghui Biotechnology (Hunan, China). KGN cells were cultured in DMEM/F12 medium (Hyclone, USA) supplemented with 10% FBS (ExCell Bio, China), 100 IU/mL penicillin and 100 mg/mL streptomycin (Gibco, USA), and incubated at 37 °C in a 5% CO_2_, 95% humidified atmosphere. For the distinction of high and low density, we followed the previous report [20]. In short, at low-density, cells existed as single cells or small colonies (3 × 10^3^ cells/cm^2^). For high-density conditions allows cells to fuse and contact with each other (1.5 × 10^5^ cells/cm^2^). The microscopy images were acquired at 72 × 72 dpi resolution using an inverted microscope (Olympus, DP80, Japan). There was no any processing to enhance the resolution of the microscopy images.

### Harvest of hUCMSC conditioned medium

The hUCMSCs at passage 3–6 were cultured in 175 cm^2^ flask in 20 mL DMEM/F12 supplemented with 10% FBS, then collected medium every 24 h until the cell density reached 90%. Subsequently, the conditioned medium collected was filtered through a 0.22 μm syringe filter and stored at − 80 °C until use. HUCMSCs conditioned media represented as UCMSCs-CM in this study.

### Cell growth curve analysis

For growth curve analysis, cells were seeded in 24 well plates with complete growth medium or UCMSCs-CM at a density of 5 × 10^3^ cells per well. Three well cells from each group were counted with a hemocytometer after 0.05% trypsin digestion every 24 h, and then generated a growth curve.

### Cell viability assay

Cell Counting Kit-8 (CCK-8, Abmole, USA) was used to detect the viability of KGN cells. KGN cells were seeded in 96 well plates and cultured for 24 h. The culture medium was changed to 100 μL fresh complete growth medium or UCMSCs-CM and cultured for 48 h, added 10 μL CCK8 solutions in each well and then cultured for 2 h. The optical density values were measured by microplate reader (Bio-Rad, 168–1130 iMark, USA) at 450 nm.

### Flow cytometry analysis

Flow cytometry was performed as previously described with some modifications [28, 29]. Apoptosis was detected by using an Annexin V-FITC apoptosis detection kit (BD Biosciences, USA). Cell cycle was detected by propidium iodide (PI) (50 μg/mL) staining. The results were assessed by FACS Aria flow cytometer (BD Biosciences, BD FACSAria^TM^III 03,141,313, USA).

### TUNEL assay

TUNEL assay was performed as previously described with some modifications [30]. After be treating with UCMSCs-CM for 48 h, the KGN cells were fixed with 4% paraformaldehyde for 30 min at room temperature. According to the TUNEL kit (green fluorescence, C1088, Beyotime, China) manufacturer's instructions, TUNEL test solution was added to the cells devoid of light at 37 °C for 60 min, and then the nuclei were finally stained with DAPI (Sigma, USA) for 10 min. The microscopy images were acquired at 72 × 72 dpi resolution using an inverted microscope (Olympus, DP80, Japan). There was no any processing to enhance the resolution of the microscopy images.

### Scratch wound assay

The motility of KGN cells was assessed by the scratch wound assay. Cells were plated into a 6-well cell culture plate at a density of 4 × 10^5^ cells/well in complete growth medium and grown to 100% confluence. Using a 200 μL pipette tip to make the straight scratch and washed with PBS twice to remove the cell debris. At 0, 24 and 48 h after the scratch, images were captured at 72 × 72 dpi resolution using an inverted microscope (Motic, AE2000, China) at × 100 magnification after incubation at 37˚C with complete growth medium or UCMSCs-CM. There was no any processing to enhance the resolution of the microscopy images.

### Cell invasion assay

The invasion abilities of KGN cells were evaluated by the matrigel invasion assay. Briefly, KGN cells in 100 μL DMEM/F12 medium containing 10 g/L HSA(CSL Behring; Australian) were plated on each 24-well transwell filter upper chamber (8 μm pore size, Corning, USA) coated with matrigel (Corning, 356,234, USA). And the lower chamber was filled with 500 μL complete growth medium or UCMSCs-CM to stimulate cell invasion. After 16 h of incubation, cells on the upper membrane surface were removed from the bottom of the filter, fixed with 4% paraformaldehyde, and stained with 5% crystal violet; 5–8 random fields of each well were captured at 72 × 72 dpi resolution under an inverted microscope (Olympus, DP80, Japan) for quantification of cell invasion. There was no any processing to enhance the resolution of the microscopy images.

### Western blot analysis

Following previous description, western blot was conducted [31]. Primary antibodies specific against cyclin D1 (60,186), p27 (25,614), Bax (50,599), Bcl-2 (12,789), β-actin (20,536) ( all from proteintech; USA), YAP1 (ab52771), p-YAP 1(S127) ( ab76252) ( all from abcam; UK), LATS1( 3477 T), p-LATS1 (S909) (9157S) ( all from Cell Signaling Technology; USA), all the antibodies were used at the concentration 1:1000. Secondary antibody was HRP-labeled donkey anti-mouse/rabbit IgG (H + L) (1:2000) (Proteintech, USA).

### Immunostaining

Immunofluorescence was performed as previously described with some modifications [32]. Cells were fixed in 4% paraformaldehyde and permeabilized with 0.02% Triton X-100 in PBS, after washing, the cells were blocked with 1% FBS at 25 °C for 20 min and then incubated with the primary antibody, rabbit polyclonal anti-YAP antibody (1:200) at 4 °C for 12 h, after washing, the cells were labeled with a secondary antibody coupled with Aleax-488(1:1000) (Abcam, ab150117, UK) at room temperature for 2 h. Cells were stained with DAPI (Sigma, USA) at room temperature for 10 min. Fluorescent images were captured at 96 × 96 dpi resolution using a confocal microscope (Nikon, TiE-A1 plus, Japan). There was no any processing to enhance the resolution of the microscopy images.

### Statistical analysis

All data were expressed as the Mean ± SEM for at least three independent experiments. Statistical analysis was carried out by GraphPad Prism 8 software (San Diego, USA). Differences among groups were evaluated by the Student's t test. *P*-values were calculated, and statistical significance is displayed as *P < 0.05, **P < 0.01. ***P < 0.001, ****P < 0.0001, NS: not significant, *P* > 0.05.

## Results

### Characterization of hUCMSCs

**Fig. 1:**
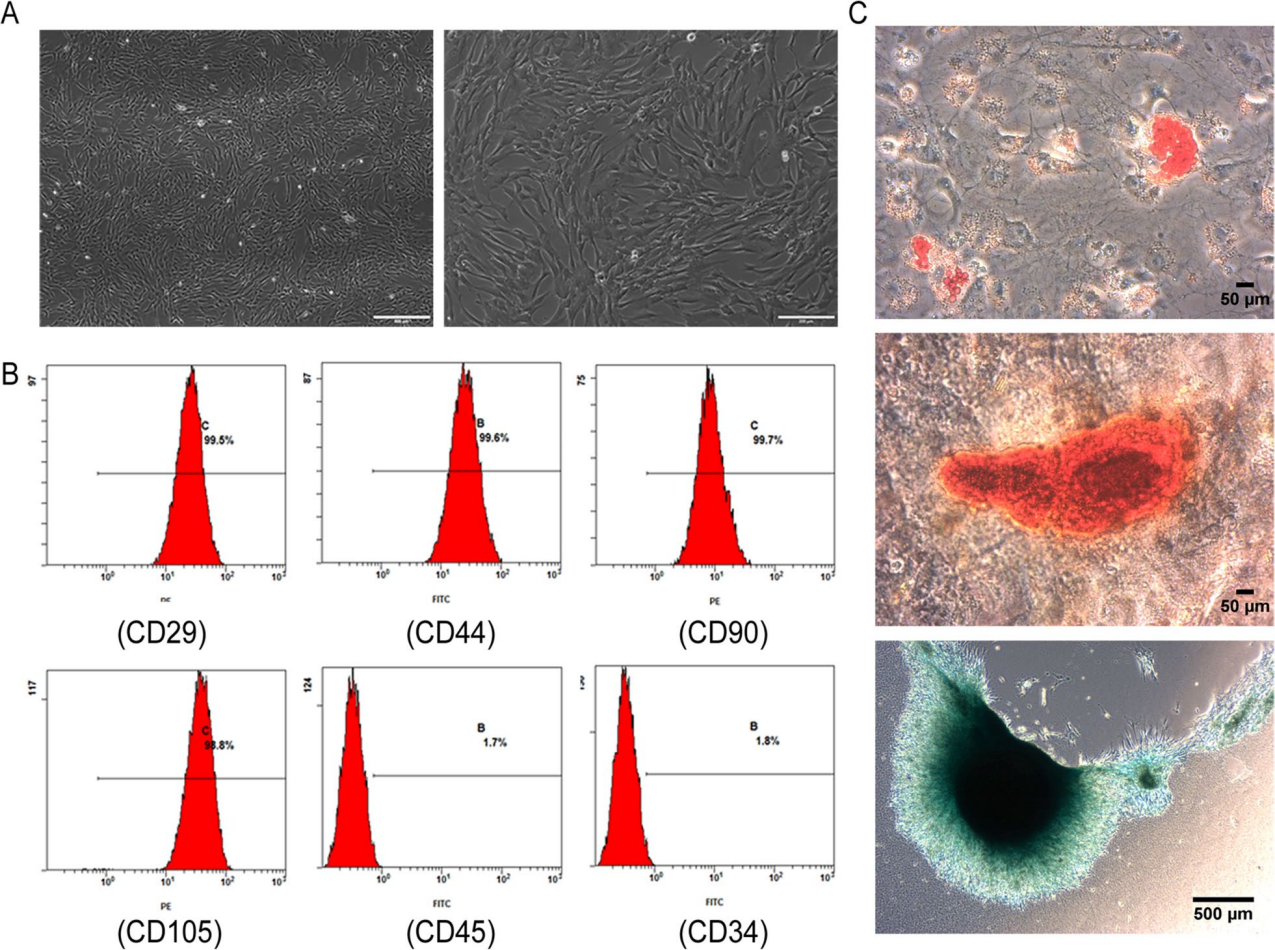
Characterization of hUCMSCs. **A** HUCMSCs exhibited fibroblast-like morphology. Scale bar, 500 μm and 200 μm. **B** Flow cytometry analysis of CD29, CD90, CD44, CD105, CD34 and CD45 expression in hUCMSCs. **C** Differentiation of hUCMSCs into adipocytes (top row). Scale bar, 50 μm; Osteocytes (middle row). Scale bar, 50 μm; Chondrocytes (bottom row). Scale bar, 500 μm

The morphology of the isolated hUCMSCs was a class of swirling and fibroblast-like cells under a light microscope (Fig. 1A). The isolated hUCMSCs expressed MSC-related CD surface markers, namely, CD29, CD90, CD44, and CD105. In addition, the hUCMSCs were negative for the hematopoietic markers, including CD34 and CD45 (Fig. 1B). Besides, the isolated hUCMSCs also have differentiation potential into adipocytes, chondrocytes, and osteoblasts (Fig. 1C).

### UCMSCs-CM inhibited the proliferation of KGN cells

**Fig. 2:**
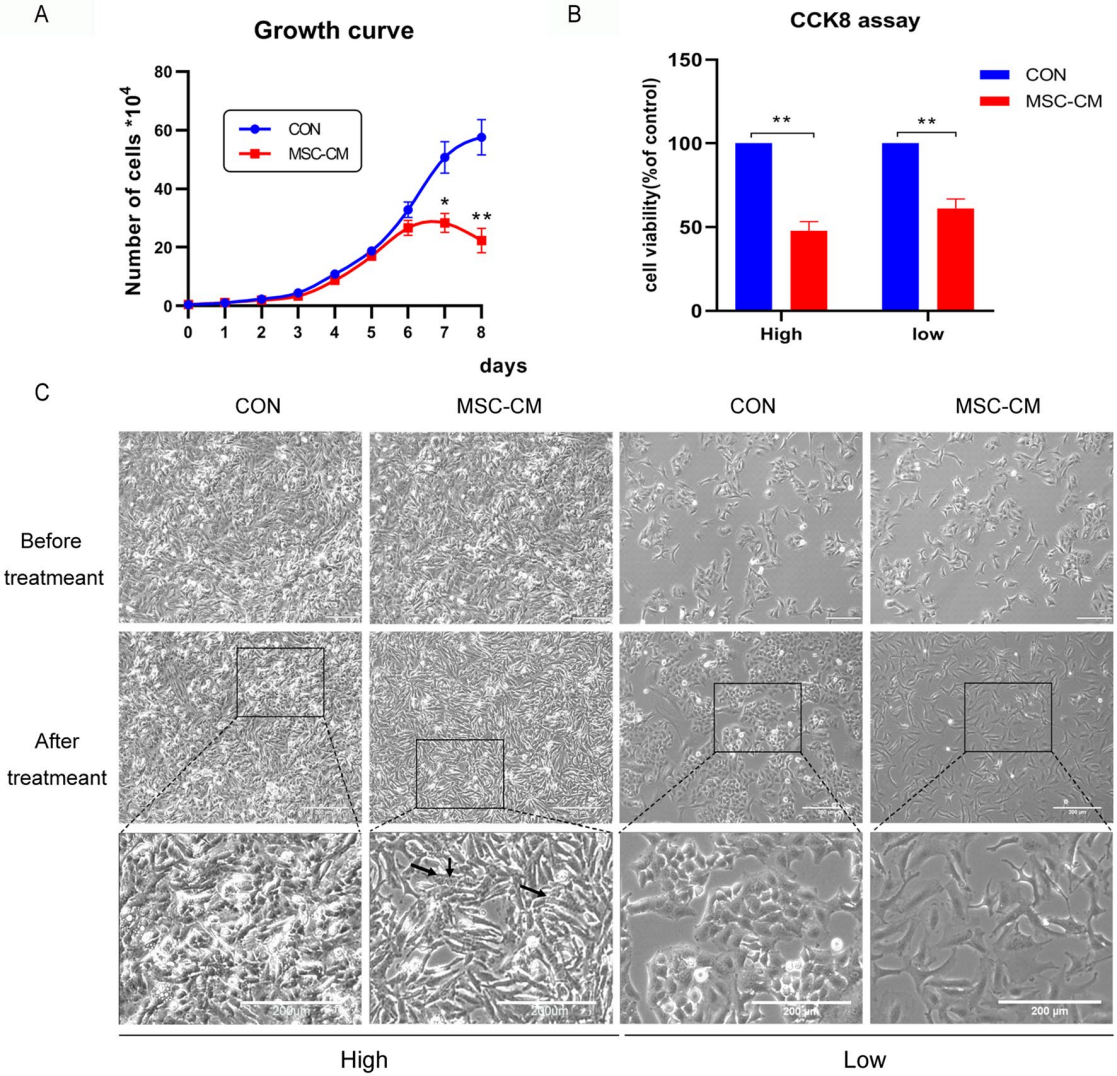
Effect of UCMSCs-CM on proliferation and cell viability of KGN cells. KGN cells were implanted at high (High) and low (Low) density. **A** Growth curve of KGN cells treated with UCMSCs-CM for 8 days. **B** CCK8 assays on KGN cells treated with UCMSCs-CM for 48 h. **C** UCMSCs-CM-induced morphological changes in KGN cells, the black arrows represent dead cells. Scale bar, 200 μm. *P < 0.05, **P < 0.01

The effect of UCMSCs-CM on the growth of KGN cells was first examined by growth curve analysis. Within 6 days after the KGN cells were implanted at 5 × 10^3^ cells/well, the cells of the control group and the UCMSCs-CM treatment group proliferated at similar rate. Noticeably, after the seventh day, at which the cells reached confluence, the cell proliferation in UCMSCs-CM treatment group slowed down significantly, while the cells of the control group continue to proliferate (Fig. 2A). To further characterize the growth inhibitory effects of UCMSCs-CM in confluent cultures of KGN cells, the cell viability of KGN cells treated with UCMSCs-CM for 48 h at low and high density were detected by CCK8 assay. The low density ensures that the contact area between cells is as small as possible, while the high density allows cells contact with each other. The result showed that the cell viability of KGN cells treated with UCMSCs-CM was decreased compared with the control group both at low and high density (Fig. 2B). In addition, whether fibroblast conditioned medium (FB-CM) exerted the same effect on high-density of KGN cells was examined, the result showed that the cell viability of KGN cells treated with UCMSCs-CM was obviously decreased compared with the control group and FB-CM group. But there was no statistical difference between the FB-CM group and the control group (Additional file 1: Fig. S1). Meanwhile, the growth and cell morphology of KGN cells was observed. There is a marked difference in the morphologic appearance of control and UCMSCs-CM treated group at high density. Cells in the control group displays a more rounded and small cell shape in dense areas, but cells in the UCMSCs-CM group rest in ellipsoid and larger structure, and cell shrinkage, membrane damage and cell death could be observed. When it at low density, compared with the control group, the density of the UCMSCs-CM group was less dense and the morphologically was altered, nevertheless, the nuclei were normal and no dead cells were observed (Fig. 2C). These data indicated that UCMSCs-CM inhibit the proliferation of KGN cells.

### UCMSCs-CM arrested the cell cycle at the G1 phase of KGN cells at high density

**Fig. 3:**
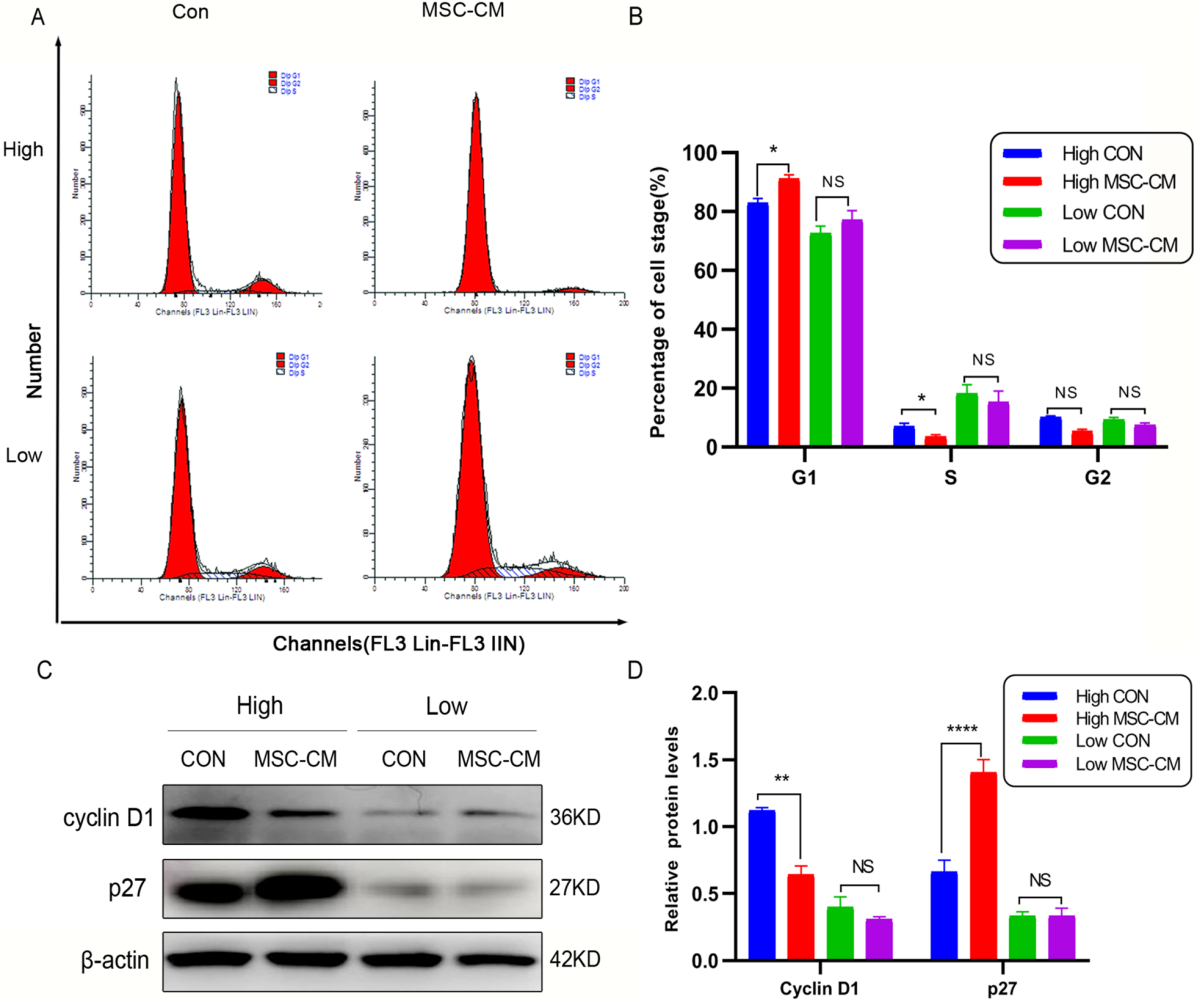
Effect of UCMSCs-CM on cell cycle of KGN cells. KGN cells were implanted at high (High) and low (Low) density. **A** Representative flow cytometry images of KGN cells treated with UCMSCs-CM for 48 h. **B** The quantitative results of flow cytometry assay. **C** The expression of cyclin D1 and p27 in KGNs detected by western blotting. Full-length blots are presented in Additional file 2: Fig. S4. **D** The quantitative results showing the relative expression levels of cyclin D1and p27 proteins. β-actin was used as loading control. *P < 0.05, **P < 0 .01, ****P < 0 .0001. *NS* No statistical difference

Flow cytometry was conducted to identify changes in the cell cycle of KGN cells treated with UCMSCs-CM for 48 h at low density and high density. The result showed that upon reaching confluence, UCMSCs-CM treated KGN cells underwent a G1 cell cycle arrest (Fig. 3A, B). In order to further explain the G1 arrest in KGN cells in response to the UCMSCs-CM treatments, some cell cycle related proteins were detected by western blot. Compared with the control group, UCMSCs-CM obviously decreased the protein level of cyclin D1 and increased the protein level of p27 in KGN cells at high density (Fig. 3C, D; full-length blots were presented in Additional file 2: Fig. S4A–C). However, there was no significant difference in the expression levels of these proteins between UCMSCs-CM treatment and no treatment when it at low density. These data indicate that UCMSCs-CM inhibits the cell cycle of KGN cells at the G1 phase at high density rather than at low density.

### UCMSCs-CM induced apoptosis of KGN cells at high density

**Fig. 4:**
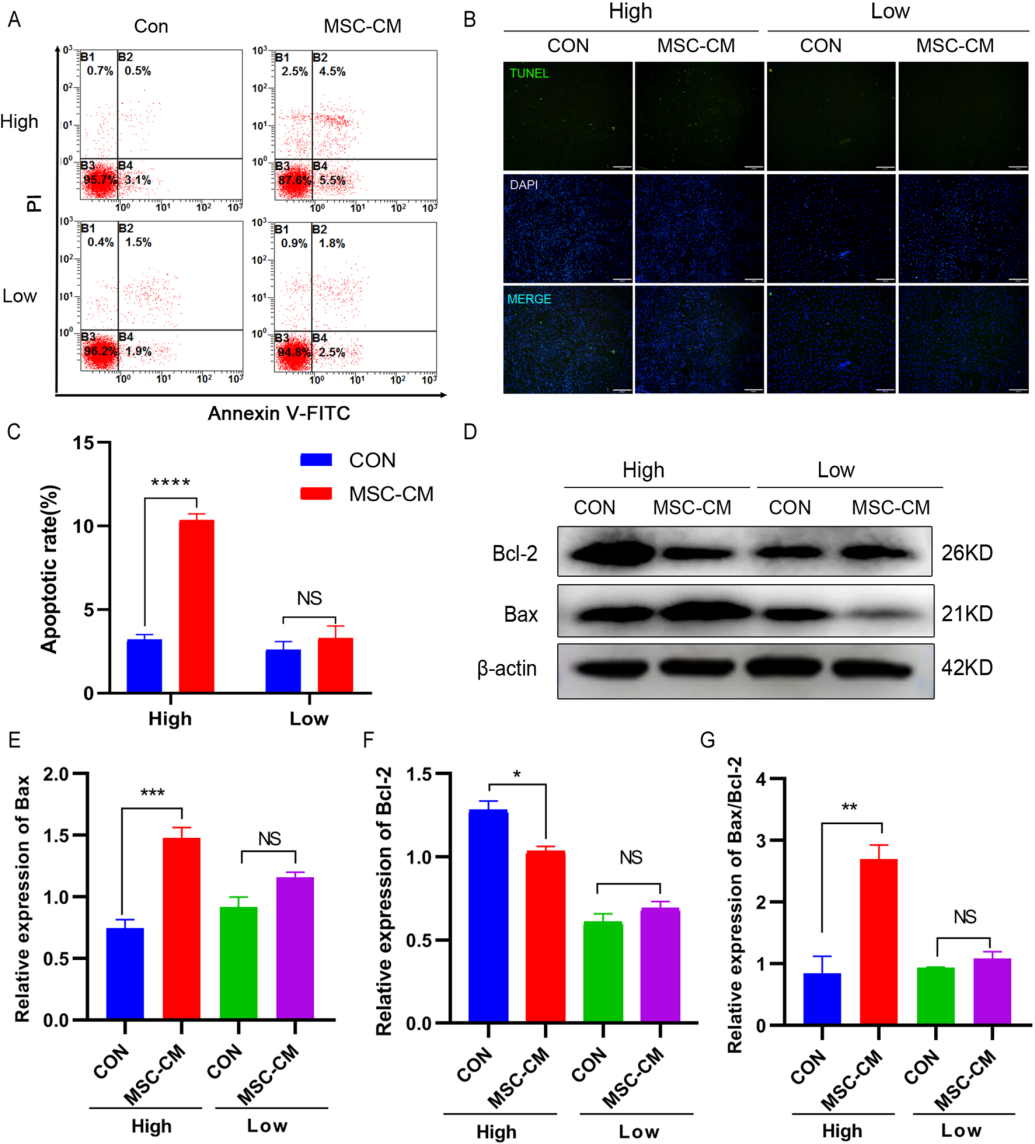
Effect of UCMSCs-CM on apoptosis of KGN cells. KGN cells were implanted at high (High) and low (Low) density. **A** Flow cytometric analysis of apoptosis in KGN cells treated with UCMSCs-CM for 48 h. **B** Representative TUNEL stain images of KGN cells subjected to UCMSCs-CM treatment. Scale bar, 200 μm. **C** The quantitative results of flow cytometry assay. **D** Expression of Bax and Bcl-2 measured by western blot. Full-length blots are presented in Additional file 2: Fig. S5. **E**–**G** The quantitative results showing the relative expression levels of Bax and Bcl-2 proteins and the ratio of Bax/Bcl-2. β-actin was used as loading control. *P < 0.05, **P < 0.01, ***P < 0.001, ****P < 0.0001. *NS* No statistical difference

The effects of UCMSCs-CM on apoptosis of KGN cells at low and high density were examined. The flow cytometry results presented UCMSCs-CM treatment led to an increase in the proportion of apoptotic cells of KGN cells at high density (10.37 ± 0.6351% vs 3.2 ± 0.5292%, *P* < 0.0001) (Fig. 4A, C). On the other hand, it had no significant effect at low density (3.3 ± 0.3667% vs 2.6 ± 0.3055%, *P* > 0.05) (Fig. 4A, C). Consistently, in the TUNEL experiment, KGN cells showed a marked increase in emitted green light fluorescence after treatment with UCMSCs-CM at high density than low density (Fig. 4B). Moreover, the levels of pro-apoptotic factors Bax was upregulated, the expression of anti-apoptotic factor Bcl-2 was downregulated and the ratio of Bax/Bcl-2 upregulated significantly in KGN cells treated with UCMSCs-CM at high density. Yet there was no apparent difference of these protein levels between UCMSCs-CM treatment and no treatment at low density (Fig. 4D–G; full-length blots were presented in Additional file 2: Fig. S5A–C). Based on these data, UCMSCs-CM could induce KGN cells apoptosis at high density while it had no obvious effect when it at low density.

### UCMSCs‑CM inhibited the migration and invasion of KGN cells

**Fig. 5:**
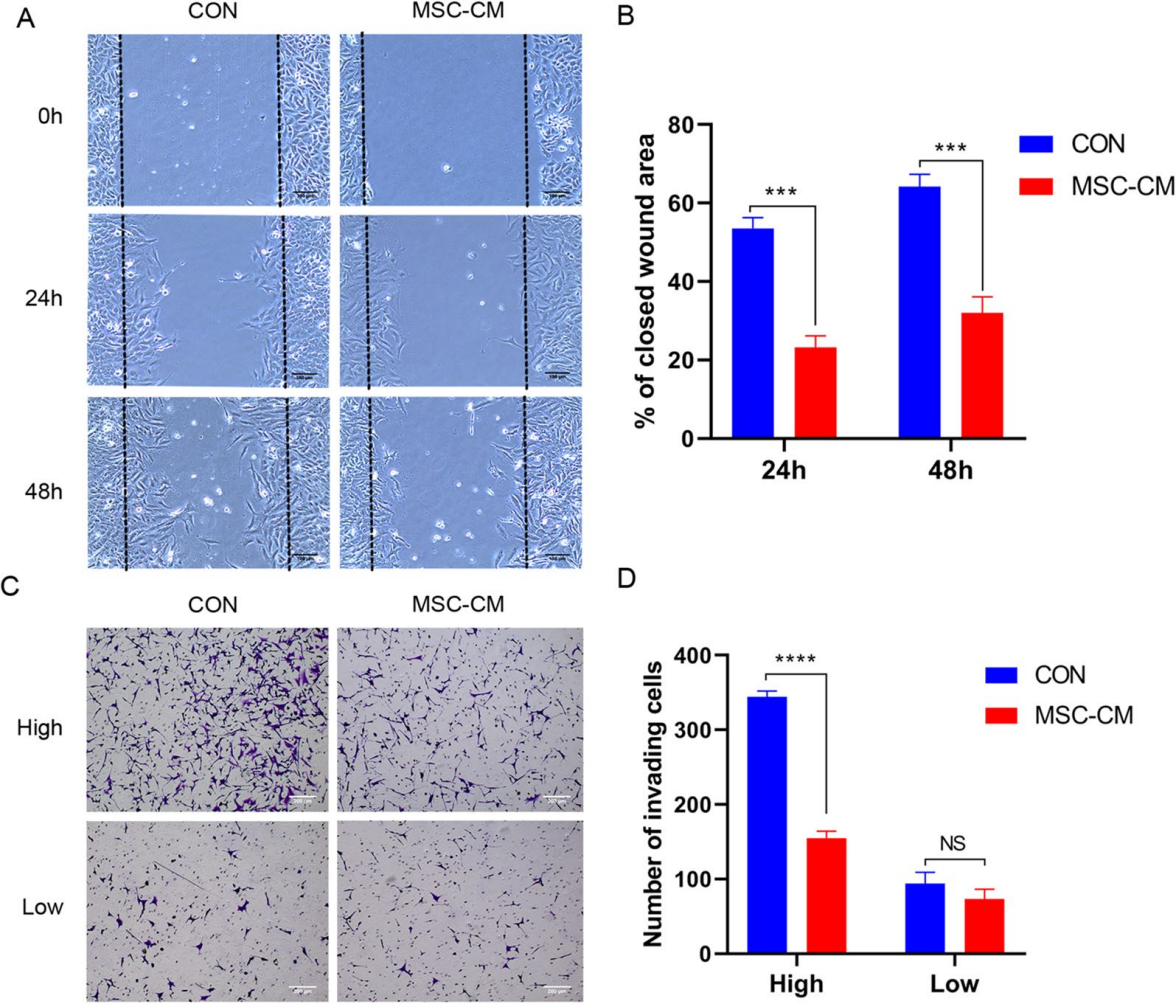
Effect of UCMSCs-CM on migration and invasion of KGN cells. KGN cells were implanted at high (High) and low (Low) density. **A** Scratch wound assays of KGN cells treated with UCMSCs-CM for 24 h and 48 h. Scale bar, 200 μm. **B** Quantification of the closed wound area for KGN cells. **C** Representative images of invasion assays. Scale bar, 200 μm. **D** The quantitative results of invading cell numbers. ***P < 0.001, ****P < 0.0001. *NS* No statistical difference

The migration of KGN cells treated with UCMSCs-CM was next assessed by performing scratch wound assays. Compared with control group, UCMSCs-CM induced a significant decrease in KGN cell migration after 24 h and 48 h, with a reduction of about 15% and about 20% in their migration distance (Fig. 5A, B). To test the effect of UCMSCs-CM on the capability of KGN cells to invade, invasion assays using matrigel-coated transwells was performed. The result showed that compared with control group, UCMSCs-CM treatment impaired KGN cell invasion as shown by the significant reduction of about 50% in the number of invading cells at high density. On the contrary, it had no effect when it at low density (Fig. 5C, D).

### UCMSCs-CM activated the Hippo-YAP signaling in KGN cells at high density

**Fig. 6:**
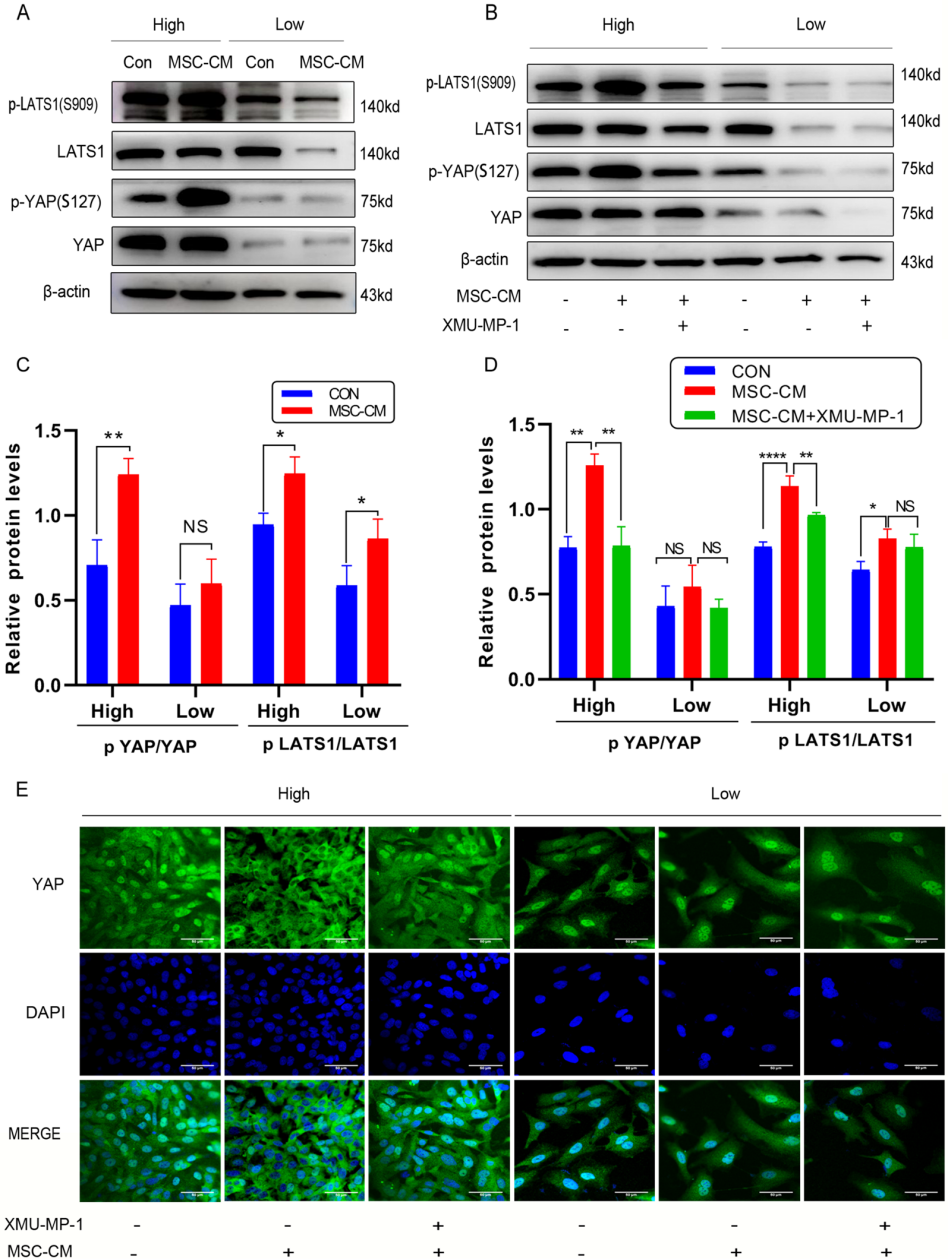
UCMSCs-CM activated the Hippo signaling in KGN cells at high density. KGN cells were implanted at high (High) and low (Low) density. **A** Expression of p-LAST1 (S909), LAST1, p-YAP (ser127), and YAP detected by western blot analysis. Full-length blots are presented in Additional file 2: Fig. S6. **B** KGNs pretreated with XMU-MP-1 and cultured with UCMSCs-CM treatment for 48 h. Expression of p-LAST1 (S909), LAST1, p-YAP (ser127), and YAP detected by western blot analysis. Full-length blots are presented in Additional file 2: Fig. S7 **C**–**D** The quantitative results showing the ratio of p-LAST1/ LAST1 and p-YAP (ser127)/YAP. **E** Representative immunofluorescence images of KGN cells stained with anti-YAP (green), DAPI (blue). Scale bar, 50 μm. β -actin was used as loading control.*P < 0.05, **P < 0.01, ****P < 0.0001. *NS* No statistical difference

Owing to the pivotal role the Hippo pathway plays in GCT development and contact inhibition [19, 26], whether UCMSCs-CM could affect this signaling pathway was examined. KGNs cells with or without treatment of UCMSCs-CM were used to analyze the expression and phosphorylation status of Hippo pathway proteins. Compared with the control group, UCMSCs-CM treatment increased phosphorylation levels of LAST1 (S909), and YAP (S127) in KGN cells at high density (Fig. 6A, C). However, there was no apparent difference in phosphorylation levels of these proteins between UCMSCs-CM treatment and no treatment at low density except LAST1 (Fig. 6A, C; full-length blots were presented in Additional file 2: Fig. S6A–E).To further validate UCMSCs-CM activated the Hippo signaling, KGN cells were pretreated with the XMU-MP-1(5 μM), which is a selective inhibitor of MST1/2, the core molecule of the Hippo pathway, for 3 h before exposure to UCMSCs-CM. Western blot analysis revealed that XMU-MP-1 effectively inhibited the phosphorylation of LAST1 and YAP induced by UCMSCs-CM (Fig. 6B, D; full-length blots were presented in Additional file 2: Fig. S7A–E). Since YAP nuclear localization is required for YAP cotranscriptional activity [24, 33–35], YAP localization in KGN cells treated with UCMSCs-CM at low density and high density was observed by confocal microscopy. As expected, when KGN cells were at low density, YAP was preferentially localized in the nucleus regardless of UCMSCs-CM or XMU-MP-1 treatment (Fig. 6E). Oppositely, when KGN cells were at high density, YAP had a dramatic cytoplasmic translocation after treatment with UCMSCs-CM. While when KGN cells were pretreated with XMU-MP-1, YAP still localized in the nucleus even after UCMSCs-CM treatment (Fig. 6E). These results indicated that UCMSCs-CM could activate the Hippo pathway in KGN cells when it at high density.

### UCMSCs-CM suppressed the malignant phenotype of KGN cells depended on activating the Hippo pathway

**Fig. 7:**
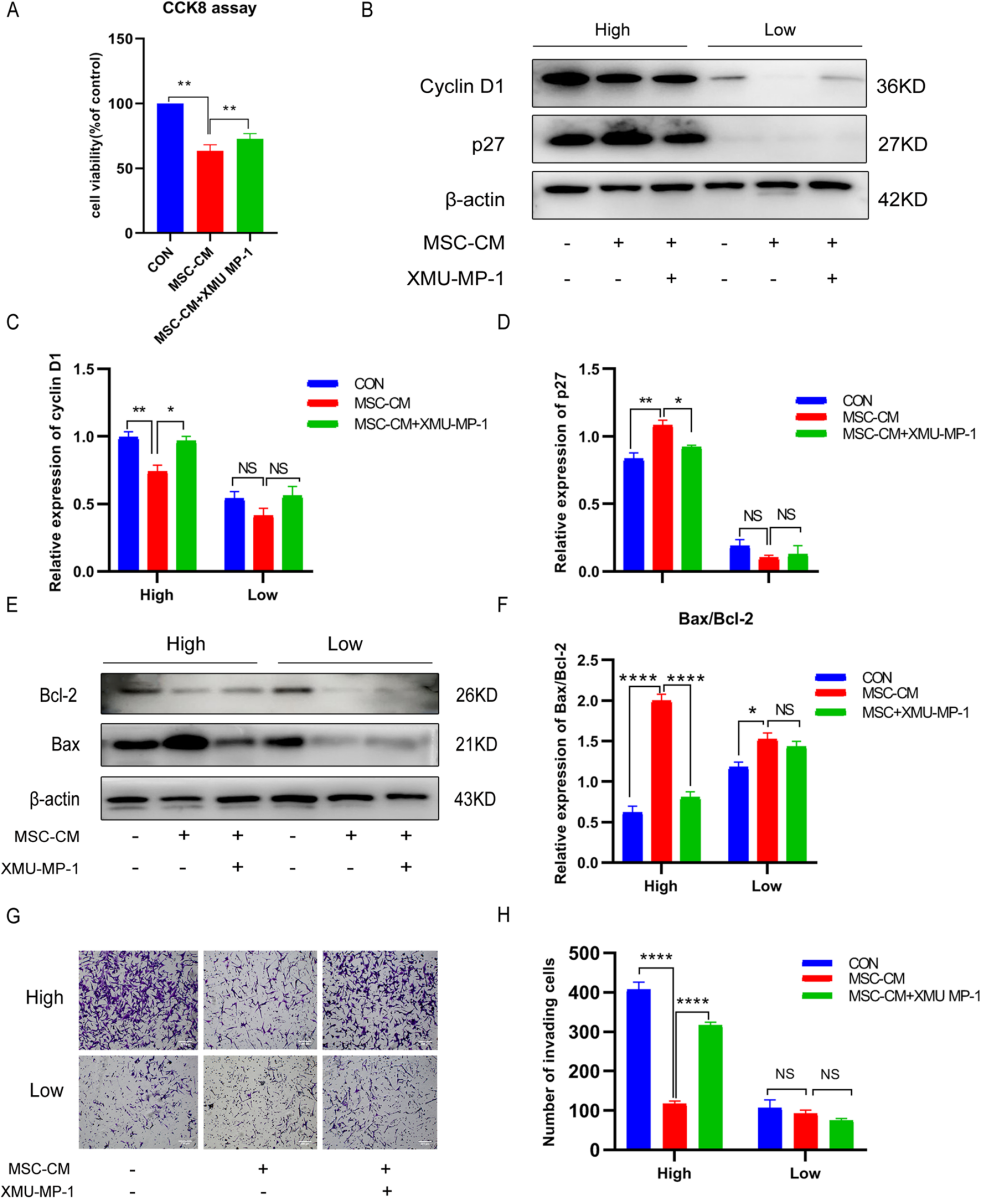
UCMSCs-CM suppresses the malignant phenotype depend on activating the Hippo pathway. KGN cells pretreated with 5 μM XMU-MP-1 and cultured with UCMSCs-CM for 48 h at high (High) and low (Low) density. **A** The cell viability was examined by CCK-8 assay. **B** Expression of cyclin D1 and p27 detected by western blot. Full-length blots are presented in Additional file 2: Fig. S8. **C** The quantitative results showing the relative expression levels of cyclin D1. **D** The quantitative results showing the relative expression levels of p27. **E** Expression of Bax and Bcl-2 detected by western blot. Full-length blots are presented in Additional file 2: Fig. S9. **F** The quantitative results showing the ratio of Bax/Bcl-2. **G** Representative images of invasion assays. Scale bar, 200 μm. **H** The quantitative results of invading cell numbers. *P < 0.05, **P < 0.01, ****P < 0.0001. *NS* No statistical difference

Since UCMSCs-CM activated the Hippo pathway in KGN cells at high density, did UCMSCs-CM inhibit the malignant phenotype of KGN cells depend on the Hippo pathway? KGNs were pretreated with XMU-MP-1 before UCMSCs-CM treatment, the results showed that XMU-MP-1 suppressed UCMSCs-CM-induced decline of cell viability (Fig. 7A). Additionally, XMU-MP-1 treatment also ameliorated the changes of the expression levels of cyclin D1 and p27 induced by UCMSCs-CM (Fig. 7B–D; full-length blots were presented in Additional file 2: Fig. S8A–C). These results indicated that UCMSCs-CM suppress proliferation of KGN cells through the Hippo signaling pathway. Next, whether UCMSCs-CM-mediated promotion of apoptosis in KGN cells could be reversed by XMU-MP-1 was examined by western blot analysis, the result revealed that XMU-MP-1 treatment led to a significant reversal of the expression of apoptosis-related proteins including Bax and Bcl-2 induced by UCMSCs-CM (Fig. 7E, F; full-length blots were presented in Additional file 2: Fig. S9A–C). Furthermore, XMU-MP-1 treatment could rescue KGN cells from UCMSCs-CM-induced invasive inhibition (Fig. 7G, H). These data suggested that the inhibitive effect of UCMSCs-CM on the malignant phenotype of KGN cells was dependent on the activation of the Hippo pathway.

## Discussion

GCTs are characterized by ovarian enlargement, excessive estrogens secretion, long natural history and their tendency to recur years after initial diagnosis [36]. The KGN cell line, which derived from recurrent metastatic GCT, is the only one established cell line, and it is widely used to study GCT [37].

In recent years, MSCs have become the focus of tumor biotherapy research. However, the tumor-suppressing or tumor-promoting effects of MSCs are still the subject of controversy. The main discrepancies may be due to different types of MSCs from different sources [38–40]. One of the factors contributing to oncogenesis is that MSCs could transform into tumor-associated fibroblasts(TAF) which plays an important role in tumor development [41]. It has been reported that hUCMSC do not transform to TAF in breast and ovarian cancer unlike bone marrow mesenchymal stem cells (BMSC) [42]. However, the application of MSCs transplantation is still formations [43, 44]. Hence, MSCs-conditioned medium, which contains numbers soluble molecules including exosomes and cytokines secreted by MSCs could be used as cell-free tumor therapy [45].

In the current study, before KGN cells proliferated into confluence, UCMSCs-CM had no effect on cell proliferation. However, once the cells proliferate to contact with each other, UCMSCs-CM significantly inhibited proliferation (Fig. 2A). We hypothesized that UCMSCs-CM inhibited proliferation of KGN cells related to cell density, and UCMSCs-CM might play an anti-tumor role by restoring tumor cell contact inhibition. Then the CCK8 assay was conducted to detect the cell viability and the morphological were observed at the meanwhile. The results showed that UCMSCs-CM decreased the cell viability of KGN cells both at high and low density (Fig. 2B). However, the morphological changes of KGN cells were quite different between high and low density, the cells of the UCMSCs-CM treatment group at high density showed ellipsoid, larger structure, atrophy, and membrane damage (Fig. 2C), similar morphological changes have been previously reported due to the restoration of contact inhibition [46]. When it at low density, the UCMSCs-CM treated cells showed normal nucleus though the morphological changed a lot. It is suggested that the sensitivities of KGN cells with different densities to UCMSCs-CM were different.

More importantly, when KGN cells were implanted at high density, UCMSCs-CM could induce cell cycle arrest at G1 phase, promote apoptosis, and inhibit cell migration and invasion. However, UCMSCs-CM had no similar effect on KGN cells implanted at low density. The cell cycle of contact inhibited cells is often arrested in G1 phase [47], accompanying by the expression level changes of cyclin D1 and p27, which were reported to take part in mediating contact inhibition[48, 49]. In our study, UCMSCs-CM treatment down-regulated the expression of cyclin D1, up-regulated the expression of p27 in KGN cells when it implanted at high density. In addition, UCMSCs-CM induced the expression of pro-apoptotic factors Bax whereas inhibited the expression of anti-apoptotic factor Bcl-2 when KGN cells were implanted at high density. It is reported similar promotive effect on apoptosis and cycle arrest in human leukemic cell line K562 treated with hUCMSCs and their extracts [50]. Besides, hUCMSC extracts are reported to inhibit ovarian cancer cell lines OVCAR3 and SKOV3 in vitro by inducing cell cycle arrest and apoptosis [10]. Our results consistent with these reports and further confirmed that UCMSCs-CM inhibited proliferation in KGN cells at high density maybe through restoring contact inhibition.

Scratch wound and matrigel invasion assay demonstrated that UCMSCs-CM could strongly inhibit KGN cells migration and invasion (Fig. 5A–D). These data suggest that UCMSCs-CM had the potential to inhibit granulosa cell tumor cell motility which can be helpful to prevent metastasis. It is reported that suppressive effects of dental pulp stem cells and its conditioned medium on development and migration of colorectal cancer cells through MAPKinase pathway [51], decidua parietalis mesenchymal stem/stromal cells (DPMSCs) and their secretome inhibit the invasive characteristics of MDA231 cells in vitro [52]. Our results consistent with these studies using MSCs against cancer cell migration and invasion.

The Hippo pathway plays a critical role in the tumorigenesis of human cancer [53]. YAP is regulated by cell density, and its inactivation plays a role in cell contact inhibition [19]. Especially, YAP highly expressed in human GCT tissues, and overexpression of YAP significantly stimulates the proliferation and migration of the GCT cell line [26]. Whether UCMSCs-CM could affect the Hippo pathway in KGN cells was further evaluated. Strikingly, the phosphorylation levels of Hippo signaling proteins LATS1 (S909), and YAP (S127) were all significantly increased and nuclear localization of YAP protein was significantly decreased in the KGN cells after UCMSCs-CM treatment at high density. However, the phosphorylation levels of these proteins were basically unchanged at low density, which attributed to the different sensitivity of the Hippo pathway to cell density. Moreover, XMU-MP-1, a selective inhibitor of MST1/2, could reverse UCMSCs-CM -induced LATS1 and YAP phosphorylation, resulting in restoring cell viability, rescuing UCMSCs-CM -induced apoptosis, cell cycle related protein changes and invasion inhibition at high density. Collectively, these results implicated that UCMSCs-CM activated the Hippo pathway to inhibit the malignant phenotype when KGN cells reach confluence. There had been similar reports that the Hippo pathway were activated in tumor cells when it at high density [20, 54]. It is reported that ferroptosis is regulated by the cellular contact and density [55]. Moreover, the YAP/TAZ activation under low density renders cancer cells sensitivity to ferroptosis [56], which may explain why the cell viability decreased and the morphological changed in the UCMSCs-CM treated KGN cells at low density in our study, this speculation needs further investigation.

There have different opinions about which type of material in the conditioned medium plays the role, Da-Won Choi et al. reported that some cytokine including Dkk-1, Dkk-3 and IGFBP-3 in conditioned medium play an antitumor role [57], Hamidreza Aboulkheyr Es et al. reported that CCL5, which is a cytokine in Human adipose derived MSCs conditioned medium impeded invasiveness and immune-suppressive characteristics of breast cancer cells[58]. On the other hand, many research reported that extracellular vesicles in the conditioned medium show anti‑tumor effect via miRNAs [59, 60], cell density-dependent miRNA such as miR-590-5p, let -7a, miR-10b has been reported to inhibited the tumorigenesis by directly targeting target genes [20, 54, 61]. To confirm what kinds of substances in the medium play the roles, we extracted exosomes derived from umbilical cord mesenchymal stem cells and detected the cell viability, migration and YAP phosphorylation level of KGN cells, the result showed that there was no significant difference between exosomes treatment and no treatment in KGN cell viability, migration and YAP phosphorylation level both when it at low and high density (Additional file 1: Fig. S2A–E; full-length blots were presented in Additional file 2: Fig. S10A–C). Based on these results, the effect of exosomes in the UCMSCs-CM was excluded, we prefer that cytokines secreted by hUCMSCs in conditioned medium play this role, the cytokine array assay was conducted to compare the different cytokine levels in UCMSCs-CM and control medium. Among 104 cytokines evaluated, adiponectin, which has been reported to have antitumor effects, had the highest secrete levels compared to control group (Additional file 1: Fig. S3A, B; full-length blots were presented in Additional file 2: Fig. S11A, B). Interestingly, it has been reported that adiponectin can inhibit tumor progression by regulating tumor cells (including ovarian cancer cells) proliferation and inducing the apoptosis, and low adiponectin have been associated with increased risk of ovarian cancer [62–64]. However, clinical trials based on administration of a single cytokine have been conducted for the treatment of different kinds of diseases, but the results have not been encouraging[65]. Praveen Kumar L et al. supposed that MSC-CM should better orchestrate a “symphony of signals” rather than what can be effected by the ad-ministration of single cytokine [66, 67]. Screen out the cytokines or combinations of cytokines that play the roles warrants further investigation and it is a long and rigorous process.

## Conclusions

**Fig. 8:**
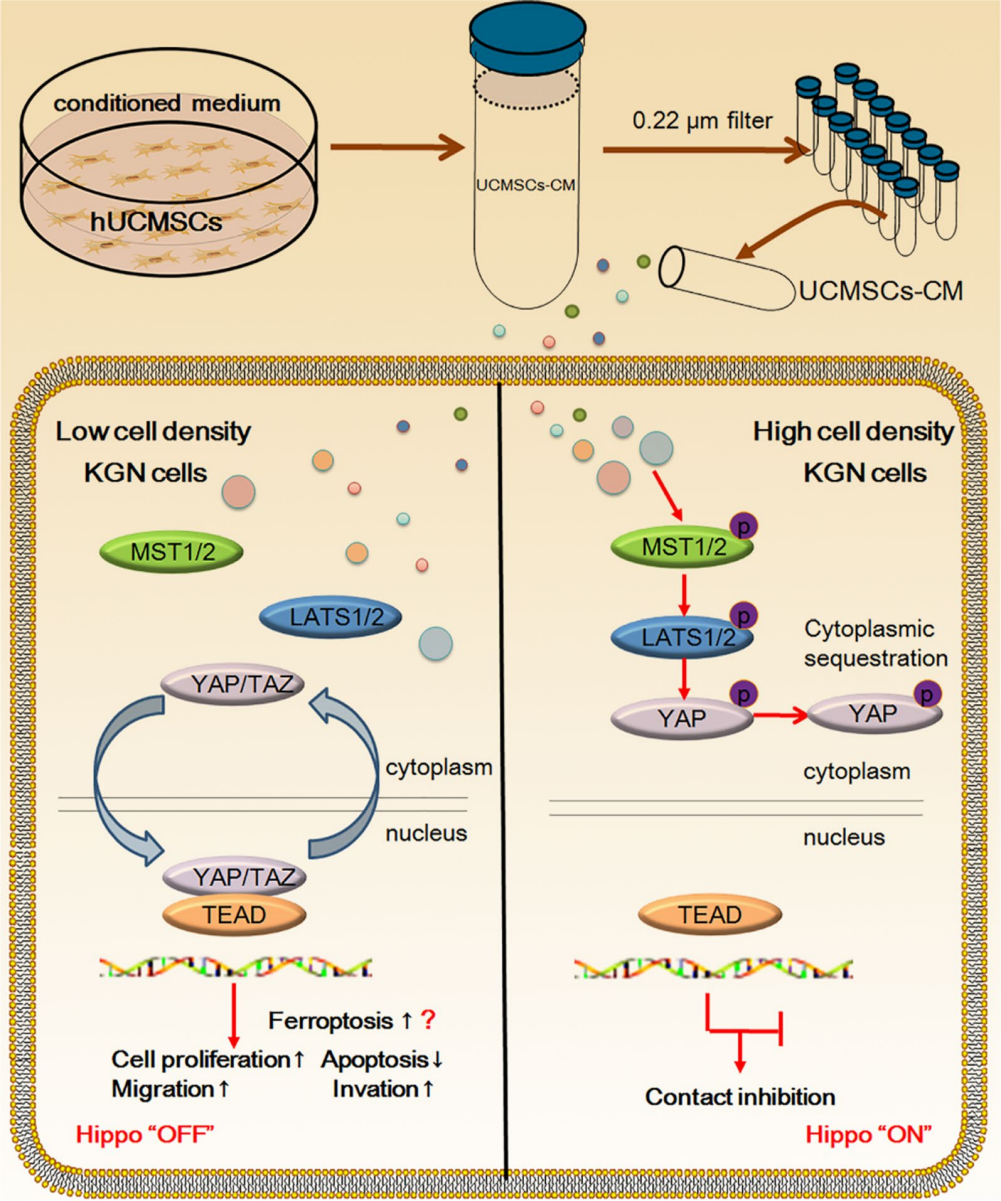
UCMSCs-CM could significantly inhibit cell viability, cell migration and invasion, induce cell cycle arrest at G1 phase and promote apoptosis of KGN cells at high density through restoration of contact inhibition of KGN cells by activating the Hippo pathway

In conclusion, UCMSCs-CM could significantly inhibit cell viability, cell migration and invasion, induce cell cycle arrest at G1 phase and promote apoptosis of KGN cells at high density through restoration of contact inhibition of KGN cells by activating the Hippo pathway (Fig. 8). These findings suggest that UCMSCs-CM is a promising therapeutic candidate for GCT treatment.

## Supplementary Information

**Additional file 1**. supplementary materials and methods and supplementary figures S1–S3.

**Additional file 2**. supplementary figures S4–S11.

## Data Availability

The data that support the findings of this study are available from the corresponding author upon reasonable request.

## Abbreviations

CCK8: Cell counting kit-8
CD markers: Cluster of differentiation markers
CM: Conditioned medium
DMEM/F12: (Dulbecco's modified eagle media, nutrient mixture F-12) (1:1)
FB: Fibroblast
GCT: Granulosa cell tumor
HSA: Human serm albumin
IgG: Immunoglobulin G
KGN: Granulosa tumor cell line
Mean ± SEM: Mean ± Standard error of mean
PBS: Phosphate‑buffered solution
UCMSCs: Umbilical cord mesenchymalstem cells
YAP: Yes-associated protein
