# Supplementary material for: Human umbilical cord mesenchymal stem cells conditioned medium exerts anti-tumor effects on KGN cells in a cell density-dependent manner through activation of the Hippo pathway
Source: Stem Cell Res Ther. 2023 Mar 20;14:46. doi: 10.1186/s13287-023-03273-z (PMC10029233; doi:10.1186/s13287-023-03273-z)
Supplement: Supplementary file 1 — Additional file 1. supplementary materials and methods and supplementary figures S1–S3. [file 13287_2023_3273_MOESM1_ESM.pdf]

Materials and methods

1. Human skin fibroblast culture and harvest of conditioned medium

Human skin fibroblast (FB) was obtained from Luoweita Biotechnology (Shaanxi, China). FB cells were cultured in DMEM/F12 medium (Hyclone, USA) supplemented with 10% FBS (ExCell Bio, China), 100 IU/mL penicillin and 100 mg/mL streptomycin (Gibco, USA), and incubated at 37°C in a 5% CO_2, 95% humidified atmosphere. The FB cells at passage 3-5 were cultured in 175 cm^2 flask in 20 mL DMEM/F12 supplemented with 10% FBS. Then collected medium every 24 h until the cell density reached 90%. Subsequently, the conditioned medium collected was filtered through a 0.22 μm syringe filter and Stored at - 80°C until use. FB conditioned media represented as FB-CM in this study.

2. Extraction of hUCMSCs exosomes

The hUCMSCs at passage 3-6 were used to extraction of exosomes. When hUCMSCs reached 80% confluence, hUCMSCs were washed twice with PBS and cultured with DMEM/F12 containing 10% exosome free serum (Vivacell, China, C3801-0100) for 48 h. The supernatant was collected and centrifuged at 2000 × g for 10min and filtered through a 0.22 μm filter. Then, the supernatant was ultra-centrifuged at 10,000 × g for 1 h and 100,000 × g for 4.5 h at 4 ℃ (HITACHI CS120FNX, Japan). The enriched exosomes were diluted in PBS and filtered through a 0.22 μm filter before stored at − 80 ℃.

3. Antibody-based cytokine arrays

The CM transferred to a centrifugal column with a 3 kDa cut-off (Millipore, Billerica,

MA, USA) following the manufacturer's instructions. Membranes from a human protein cytokine array kit (Proteome Profiler™ Array; R&D, Minneapolis, MN, USA) were used to assay relative levels of 104 cytokines in the 50 × concentrated control medium (negative control) and UCMSCs-CM according to the manufacturer's instructions.

Fig. S1

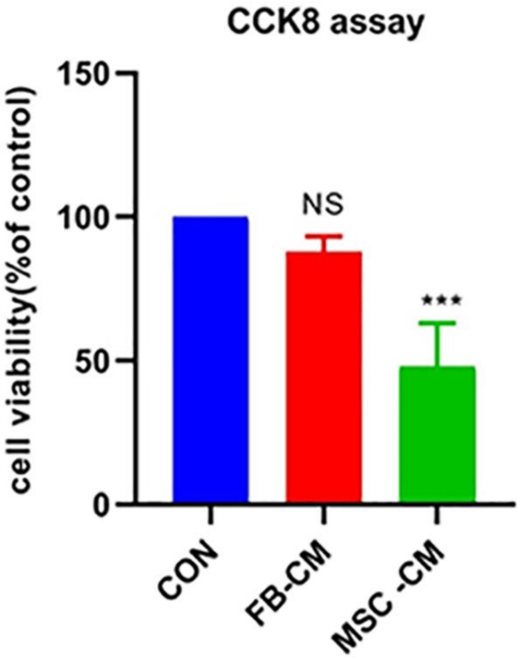

Fig. S1. Effect of UCMSCs-CM and FB-CM on cell viability of KGN cells.

Fig. S2

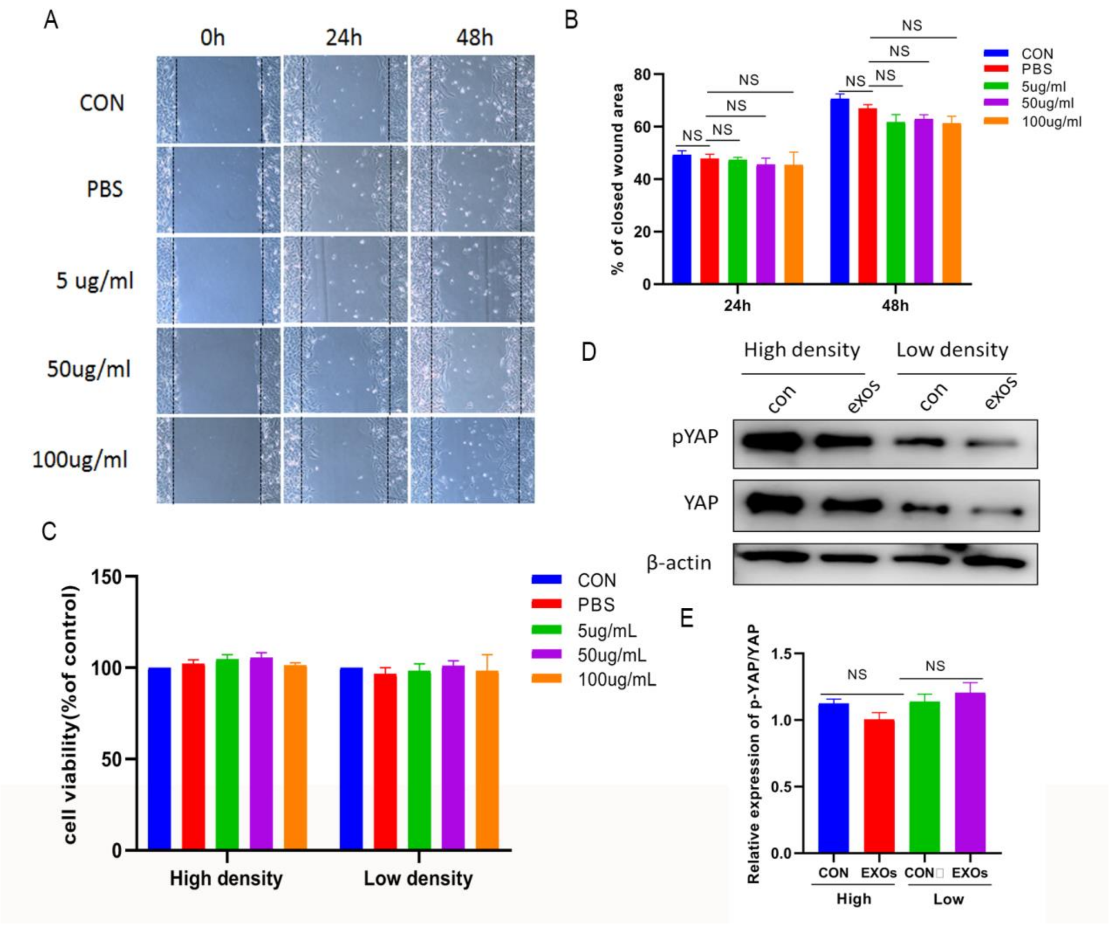

Fig. S2 Effect of exosomes on KGN cells. (A) Scratch wound assays of KGN cells treated with various concentrations of exosomes (0, 5, 50, and 100 ug/mL) for 24 h and 48 h. Scale bar, 200 μm. (B) Quantification of the closed wound area for KGN cells. (C) CCK8 assays on KGN cells treated with various concentrations of exosomes (0, 5, 50, and 100 ug/mL) for 48 h. (D) Expression of p-YAP (ser127), and YAP in KGNs with or without exosomes (100 ug/mL) treatment detected by western blot analysis. Full-length blots are presented in Fig. S10. β-actin was used as loading control. (E) The quantitative results showing the ratio of p-YAP (ser127)/YAP. $^*P < 0.05$, $^{**}P < 0.01$, $^{****}P < 0.0001$. NS, no statistical difference.

Figure S3

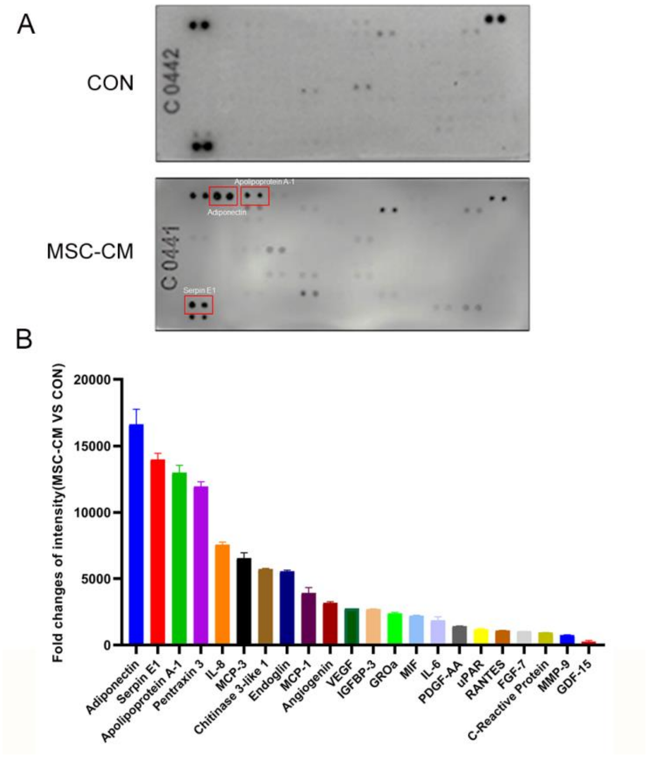

Fig. S3 Antibody-based cytokine arrays result. (A) Representative array images of antibody-based cytokine arrays. The upper is the control group, the lower is UCMSCs-CM. Adiponectin, serpin E1, apolipoprotein A-1are highlighted with red boxes. Full-length blots are presented in Fig. S11 (B) The quantitative results showing the spotted antibody array results. Data are presented as spot intensity relative to the negative control and normalized to positive control (reference spots).
