# Supplementary material for: Human umbilical cord mesenchymal stem cells conditioned medium exerts anti-tumor effects on KGN cells in a cell density-dependent manner through activation of the Hippo pathway
Source: Stem Cell Res Ther. 2023 Mar 20;14:46. doi: 10.1186/s13287-023-03273-z (PMC10029233; doi:10.1186/s13287-023-03273-z)
Supplement: Supplementary file 2 — Additional file 2. supplementary figures S4–S11. [file 13287_2023_3273_MOESM2_ESM.pdf]

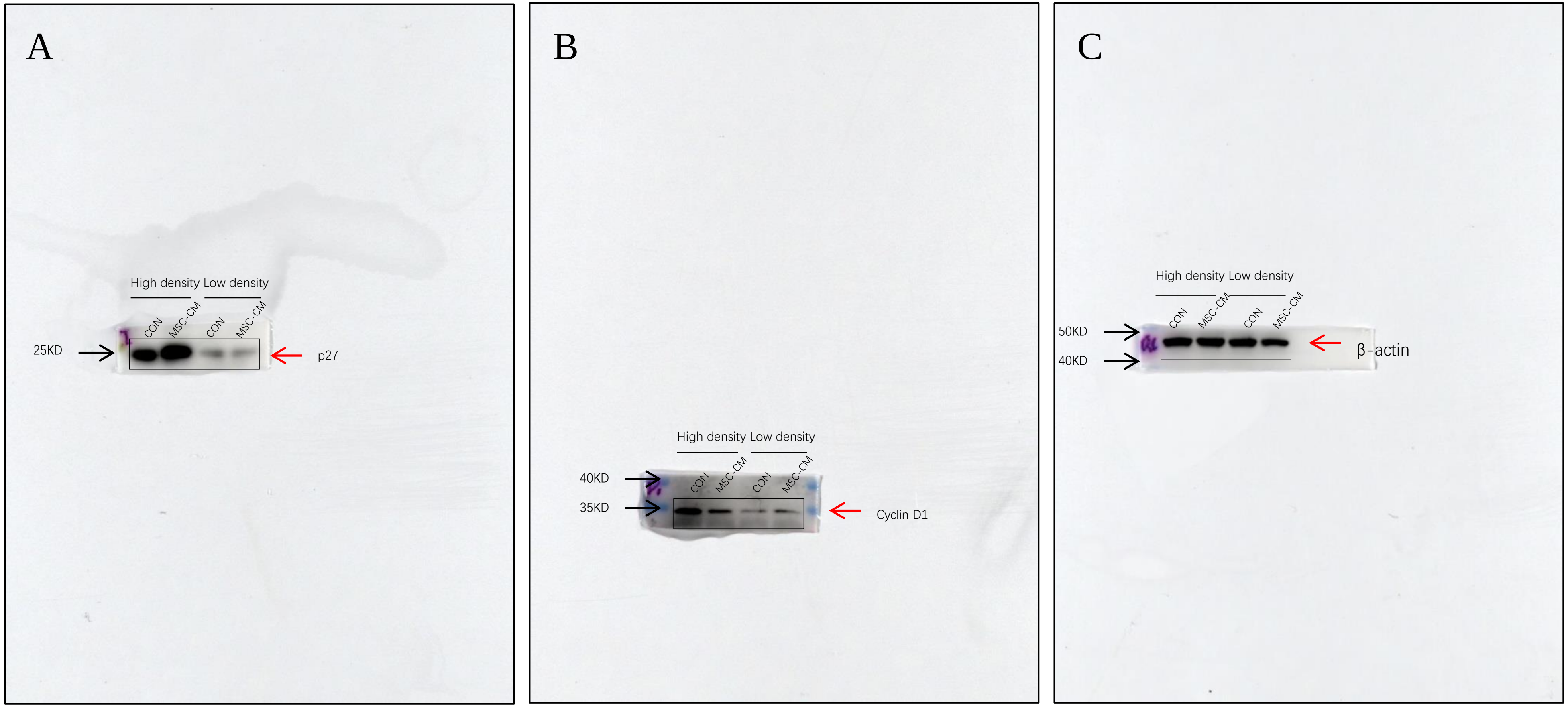

Figure S4. Full-length blots of the western blot images in figure 3C.(A: p27; B: cyclin D1; C:β-actin.)

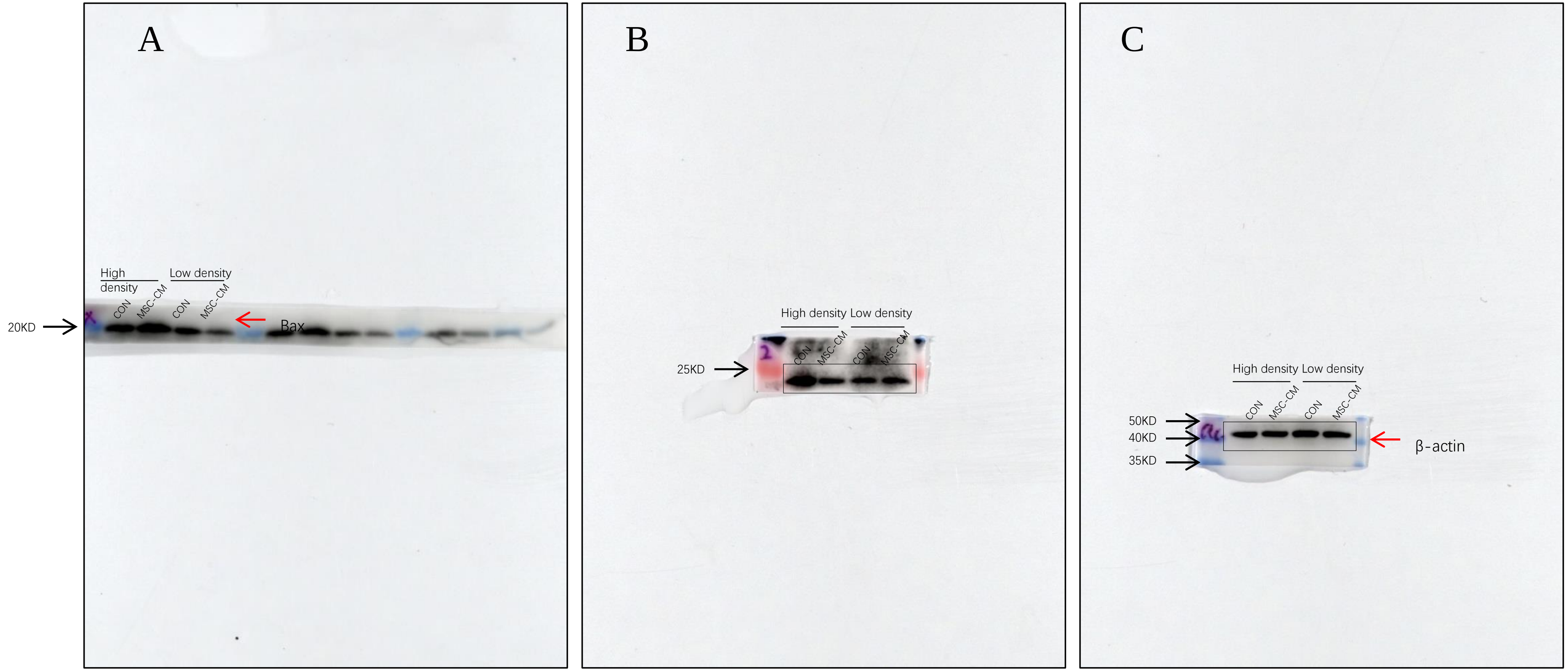

Figure S5. Full-length blots of the western blot images in figure 4D.(A: BAX; B: Bcl2; C:β-actin)

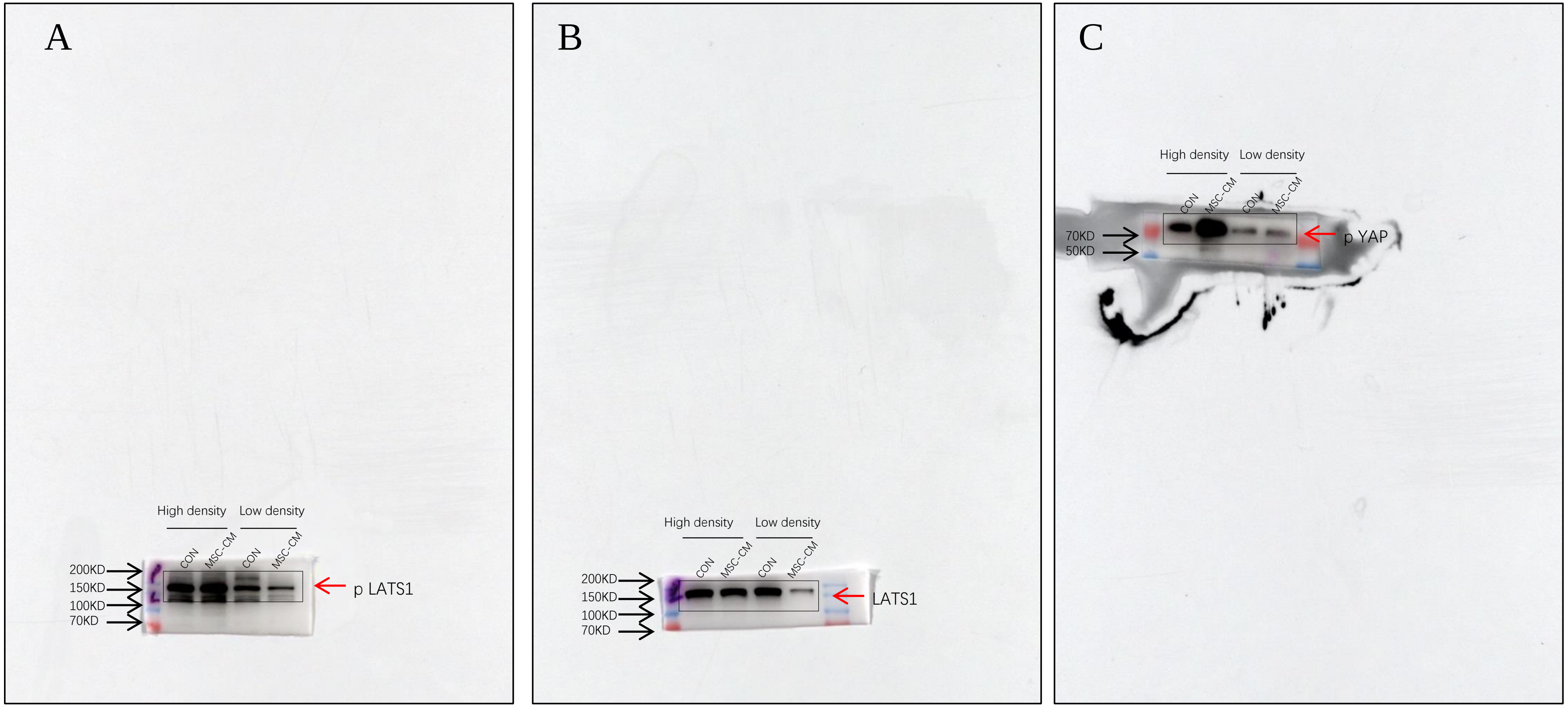
A
High density
Low density
CON
MSC-CM
CON
MSC-CM
200KD
150KD
100KD
70KD
p LATS1
B
High density
Low density
CON
MSC-CM
CON
MSC-CM
200KD
150KD
100KD
70KD
LATS1
C
High density
Low density
CON
MSC-CM
CON
MSC-CM
70KD
50KD
p YAP

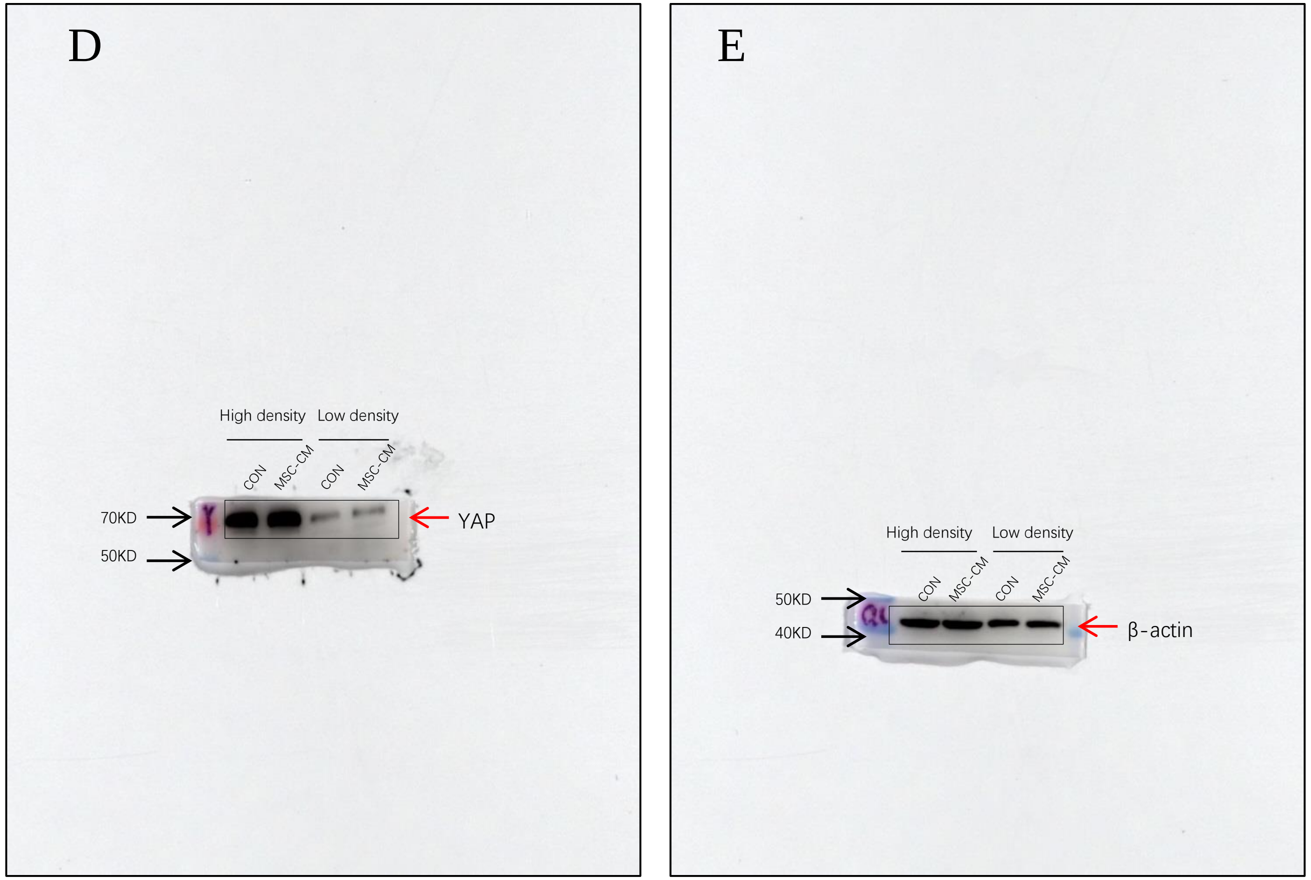

Figure S6. Full-length blots of the western blot images in figure 6A.(A:p-LATS1; B: LATS1; C:p-YAP; D:YAP; E: β-actin)

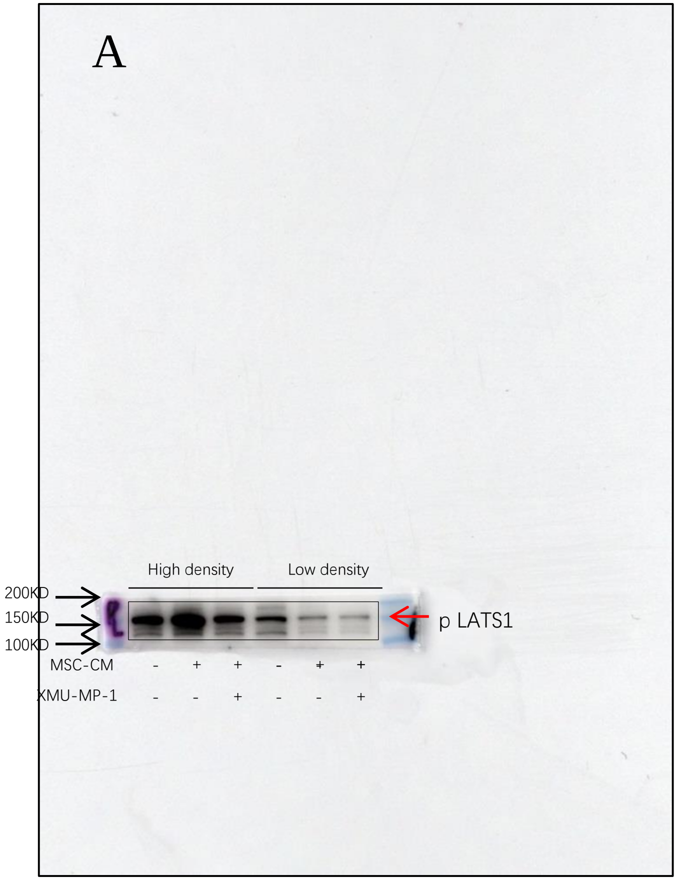
A
High density
Low density
200KD
150KD
100KD
p LATS1
MSC-CM - + + - + +
XMU-MP-1 - - + - - +

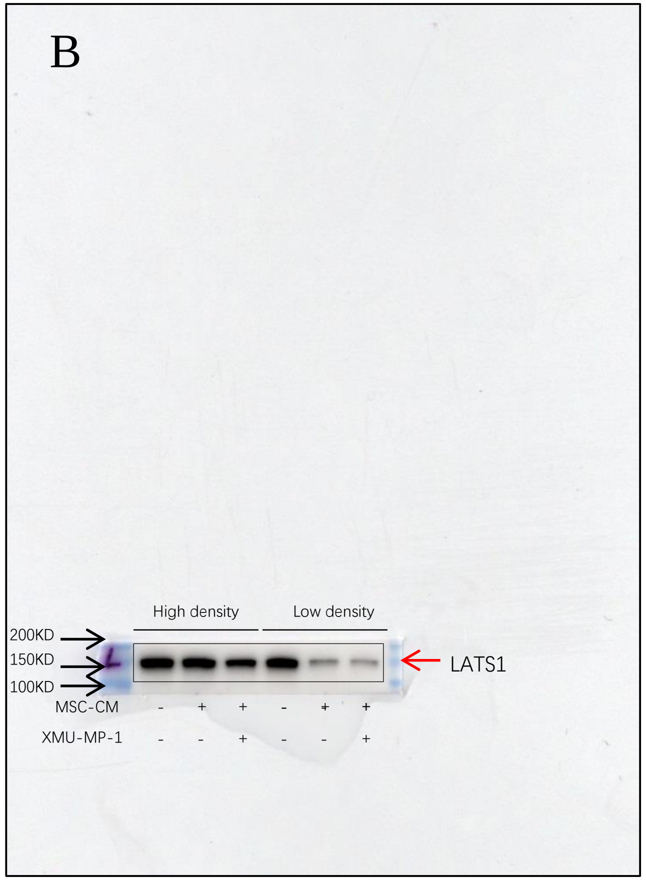
B
High density
Low density
200KD
150KD
100KD
LATS1
MSC-CM - + + - + +
XMU-MP-1 - - + - - +

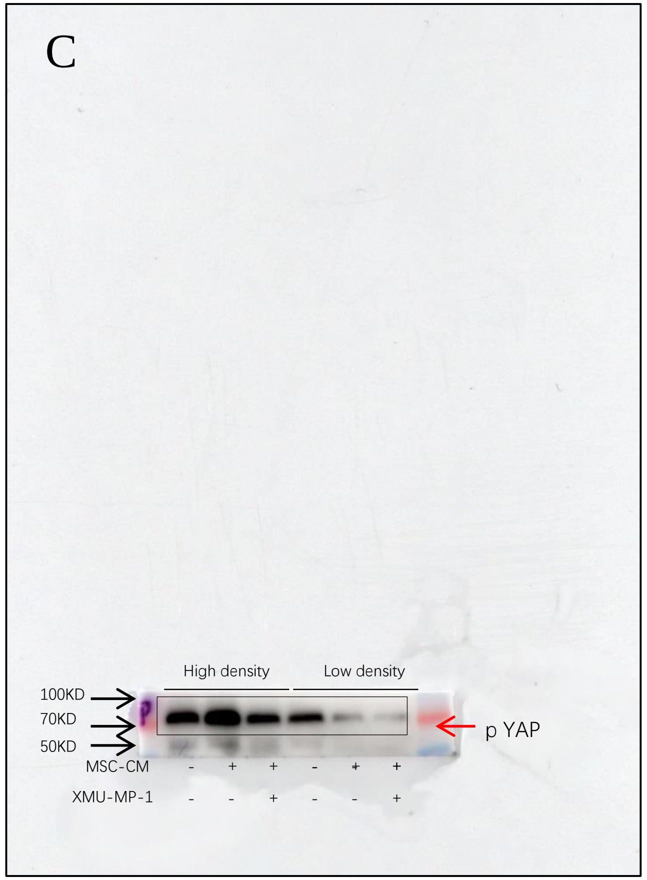
C
High density
Low density
100KD
70KD
50KD
p YAP
MSC-CM - + + - + +
XMU-MP-1 - - + - - +

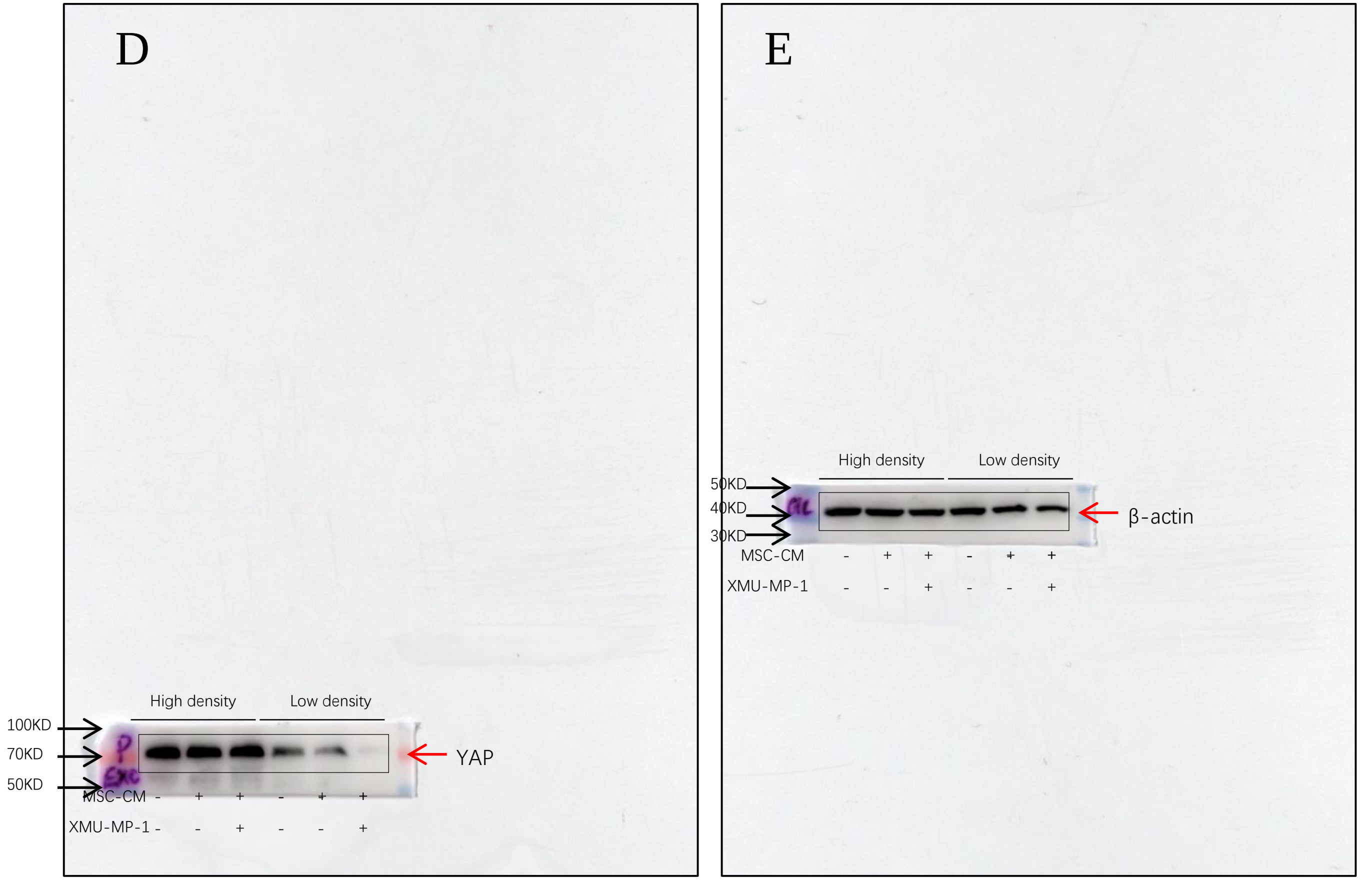

Figure S7. Full-length blots of the western blot images in figure 6B.(A:p-LATS1; B: LATS1; C:p-YAP; D:YAP; E: β-actin)

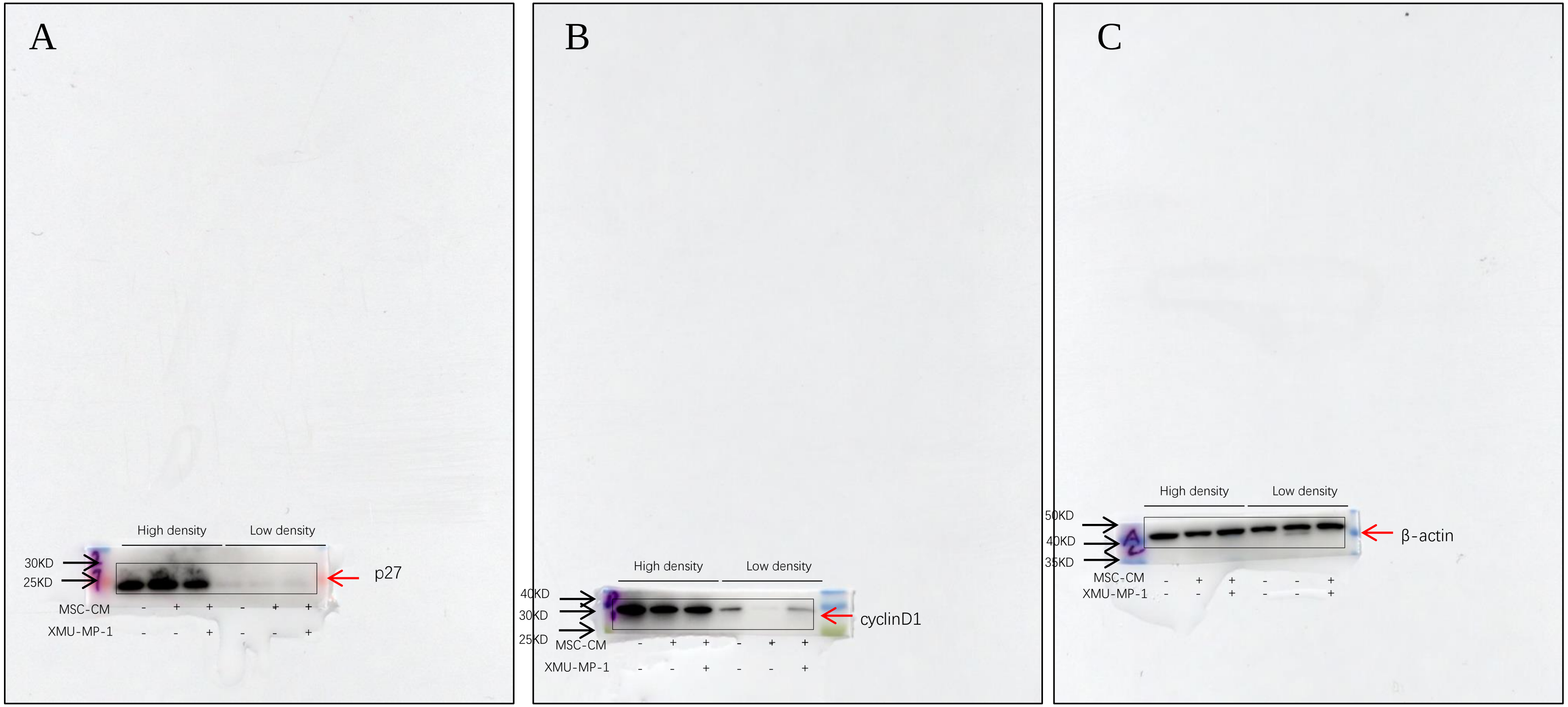

Figure S8. Full-length blots of the western blot images in figure 7B. .(A: p27; B: cyclin D1; C:β-actin.)

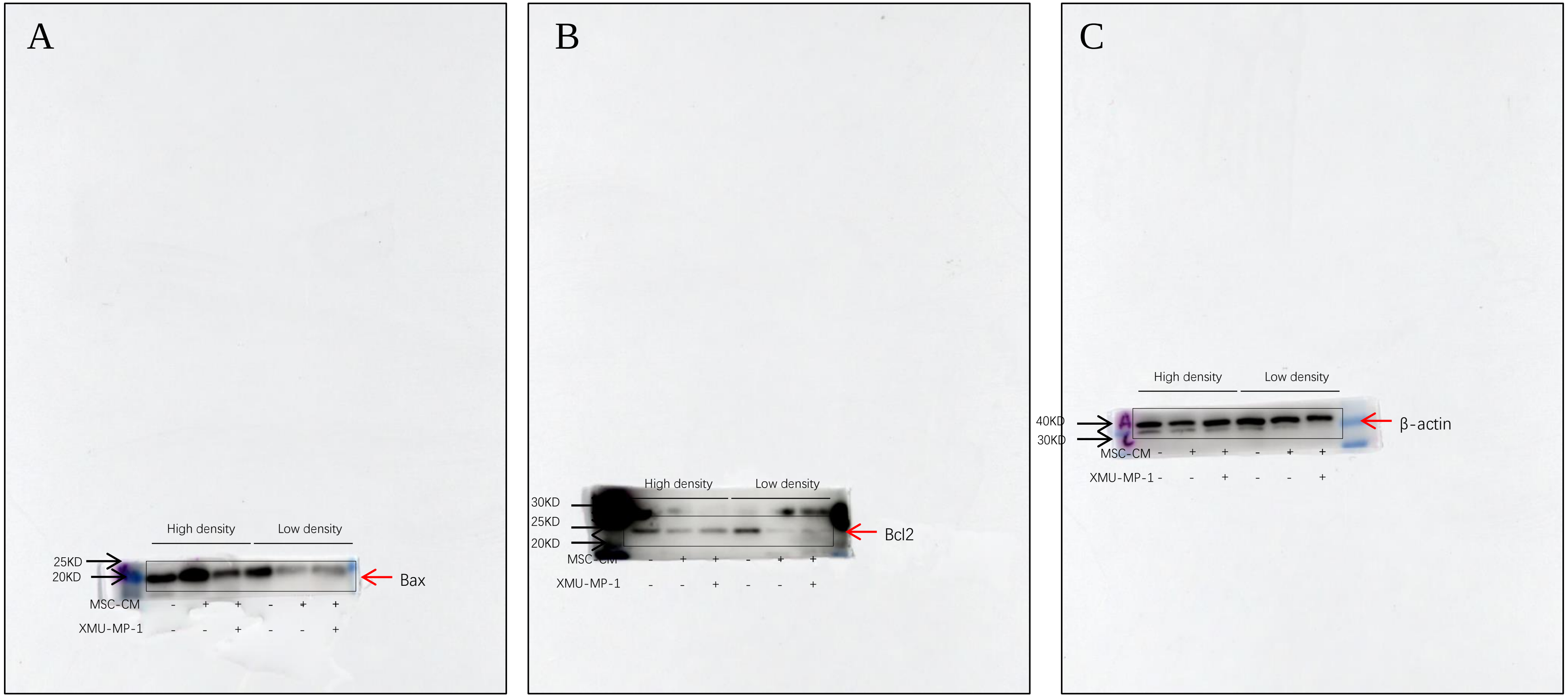

Figure S9. Full-length blots of the western blot images in figure 7E. .(A: BAX; B: Bcl2; C:β-actin)

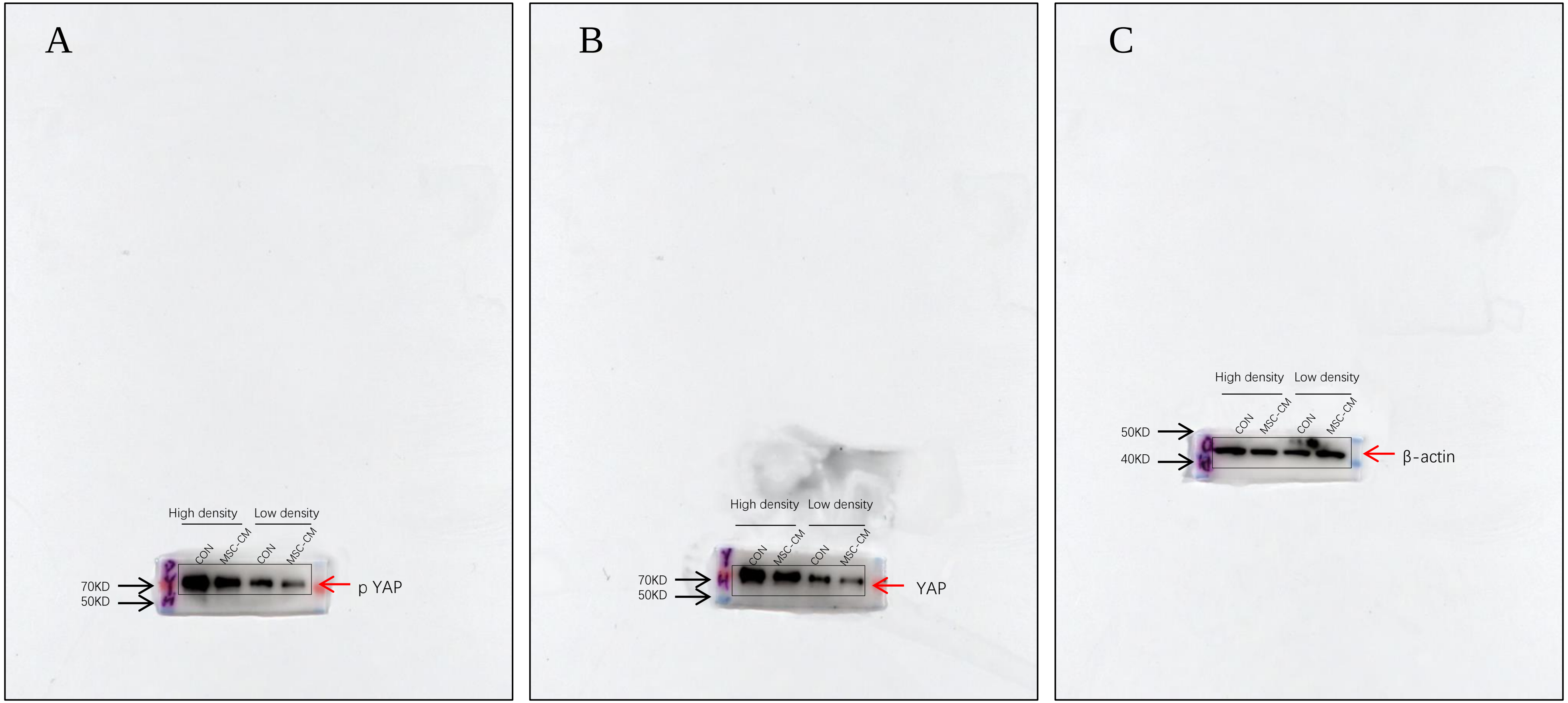

Figure S10. Full-length blots of the western blot images in figure S2D. (A: p-YAP; B:YAP; C: β-actin)

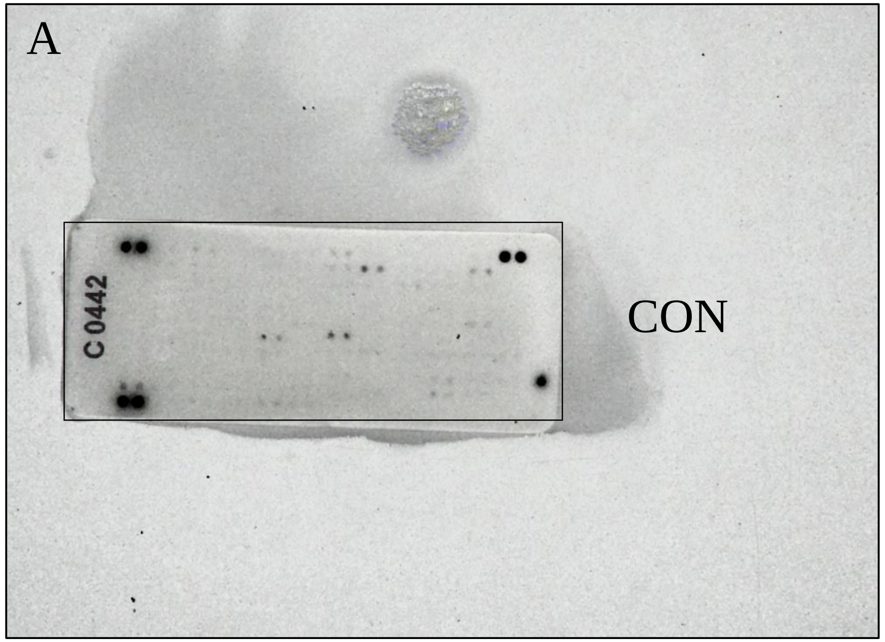

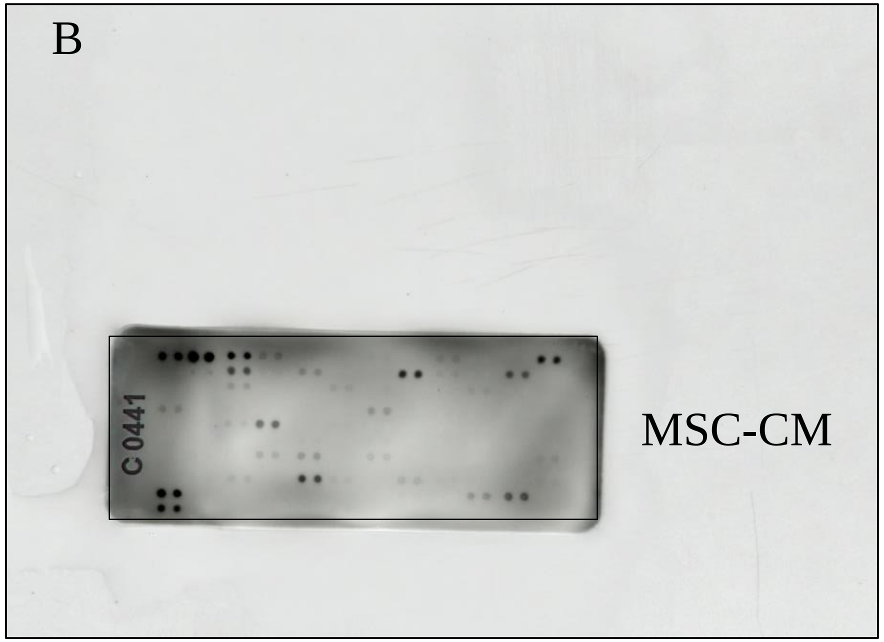

Figure S11. Full-length blots of the antibody-based cytokine arrays imagesin figure S3A.(A: CON; B:MSC-CM)
